# Single-cell multiome profiling reveals neuronal bias of modulatory role of MeCP2 phosphorylation

**DOI:** 10.1016/j.nbd.2026.107521

**Published:** 2026-07-06

**Authors:** Ziyao Zhang, Xiang Huang, Kalpana Hanthanan Arachchilage, Pramod Bharadwaj Chandrashekar, Qiping Dong, Runfeng Wang, Daifeng Wang, Qiang Chang

**Affiliations:** a University of Wisconsin-Madison, Waisman Center, Madison, WI 53705, USA; b University of Wisconsin-Madison, Department of Medical Genetics, Madison, WI 53706, USA; c University of Wisconsin-Madison, Department of Biostatistics and Medical Informatics, Madison, WI 53706, USA; d University of Wisconsin-Madison, Department of Computer Science, Madison, WI 53706, USA

**Keywords:** MeCP2, Rett syndrome, Stimulus-induced phosphorylation, Single-nuclei multi-omics, Spatial transcriptomics

## Abstract

MeCP2 is subject to many post-translational modifications, such as phosphorylation, in response to diverse stimuli. Several MeCP2 phosphorylation sites have been identified and studied individually. However, the combinatorial effect of phosphorylation at multiple sites has not been directly examined. Building on previous studies characterizing single mutations of MeCP2 phosphorylation sites, we created a mouse model carrying two mutations, serine 80 to alanine and serine 421 to glutamic acid (S80A;S421E, or A;E), in which the MeCP2 protein phosphorylation is fixed in a state observed in active neurons. A battery of behavioral tests was conducted to characterize the functional outcomes at the whole animal level, followed by two independent single-cell/nuclei level multiome profiling assays to characterize altered molecular pathways, and regulatory pattern biased towards glutamatergic neurons on both transcription and chromatin level. While integrated analysis of the multi-dimensional datasets identified cell-type specific molecular targets regulated by MeCP2 phosphorylation, the spatially resolved transcription profiling offered an orthogonal platform for target validation and provided additional information from spatial perspective. Together, results from this study revealed that the combinatorial effect of MeCP2 S80 and S421 phosphorylation is not merely synergistic, and the regulatory is biased towards glutamatergic excitatory neurons. More importantly, our study is the first to explore the single-cell multiomics profile changes in a MeCP2 phosphor mutant model and link it to the functional outcomes.

## 1. Introduction

X-linked methyl-CpG-binding protein 2 (MeCP2) plays an important yet complex role in epigenetic regulation. First identified in 1992, MeCP2 is known for its ability to recognize and bind to methylated and hydroxy-methylated cytosines (Lewis et al., 1992; Mellén et al., 2012). MeCP2 is ubiquitously expressed throughout the mammalian body and reaches the highest abundance in the central nervous system (CNS) (Shahbazian, 2002). The presence of MeCP2 is important for both neurons and non-neurons. Animal models have shown that MeCP2 is critical for neuronal synaptic numbers and strength in hippocampal glutamatergic neurons (Chao et al., 2007). In co-culture studies, MeCP2 deficient astrocytes failed to support normal neuron growth or maintain normal dendritic morphology (Ballas et al., 2009). Since DNA methylation is associated with transcriptional repression and MeCP2 can recruit co-repressor proteins to silence gene expression (Jones et al., 1998; Nan et al., 1998), it was suggested that MeCP2 functions as a repressor. However, loss of MeCP2 also leads to reduced expression of some genes, indicating that *MECP2* acts not merely as a repressor, but a transcriptional modulator that could decrease the expression of some genes and enhance the expression of others (Chahrour et al., 2008). Disruption of *MECP2* function leads to Rett Syndrome (RTT), a neurodevelopmental disorder characterized by cognitive deficits, impaired mobility, autistic- like features, seizures, and stereotyped repetitive hand movements (Amir et al., 1999; Hagberg et al., 1983; Moog et al., 2003). The mechanism through which MeCP2 could mediate such extensive and diverse regulatory effects is not completely understood.

MeCP2 is subject to diverse post-translational modifications, such as phosphorylation, in response to diverse stimuli (Damen and Heumann, 2013). Among all the phosphorylation sites, serine 421 (S421) and serine 80 (S80) are the most well studied. S421 is found to be phosphorylated upon neural activation in postmitotic neurons, where depolarization-induced Ca^2+^ influx through voltage gated calcium channels (VGCCs) (Chen et al., 2003; Zhou et al., 2006). Two calicium/calmodulin-dependent protein (CaM) kinases, CamKII and CamKIV, are implicated in mediating phosphorylation at S421 (Buchthal et al., 2012; Li and Chang, 2014; Tao et al., 2009; Zhou et al., 2006). At the functional level, loss of S421 phosphorylation causes phenotypes in hippocampal-dependent memory, enhanced LTP and synaptogenesis, increased locomotion, and altered gene expression and promoter association in the hippocampal region (Cohen et al., 2011; Li et al., 2011; Li and Chang, 2014). Opposite to the dynamics of S421 phosphorylation, S80 is the most constitutively phosphorylated residue in resting neurons and undergoes dephosphorylation upon neuron activation through VGCCs-dependent calcium influx. Homeodomain interacting protein kinase 2 (HIPK2) is found to phosphorylate S80 in vitro (Bracaglia et al., 2009). At the functional level, knock-in mice carrying the S80A dephosphorylation mutation display decreased locomotion, altered promoter association, and subtle changes in the resting neurons (Tao et al., 2009).

Although MeCP2 S421 and S80 sites bear different phosphorylation states in response to neuronal activity and lead to opposite functional outcomes, both are crucial for modulating MeCP2 protein functions. These findings raise two intriguing questions: How do these two sites collaborate and fine-tune MeCP2 protein function in vivo? Is there a combinatorial code of dynamic phosphorylation on MeCP2 function as an epigenetic regulatory module?

To explore these questions, we focused on a specific combination of MeCP2 double phosphorylation: a phosphor-dead S80 and phosphor-mimic S421 together on MeCP2, which mimics the modification status observed in activated postmitotic neurons. We generated a knockin mouse line carrying the *Mecp2*^*S80A;S421E*^ (A;E) allele in the endogenous *Mecp2* locus. We identified functional alterations in the A;E mutant mice related to baseline locomotion, open field exploration, and contextual learning and memory. Furthermore, we explored the altered molecular pathways in the hippocampus using single nuclei multiomic (snMultiomic) assays and found the impact of A;E mutation is biased towards neurons. Finally, we applied multiplexed error-robust fluorescence in situ hybridization (MERFISH) which not only serves as an orthogonal platform for validating single-cell transcriptional changes but also provided additional spatial information to the transcriptional profile. Together, results from this study generated new knowledge of the functions of MeCP2 phosphorylation. More importantly, our study is the first to provide transcription and chromatin accessibility data at the single cell resolution on a MeCP2 phosphorylation model.

## 2. Materials and methods

### 2.1. Animal model generation

Targeted construct (Li et al., 2011) including point mutations at the designed site. A T-to-G mutation was introduced in *Mecp2* exon 3 loci by PCR based mutagenesis to change serine 80 to alanine, and a TCA-to-GAG mutation in the exon 4 to change serine 421 to glutamic acid. A FLAG tag was added at the end of the *Mecp2* sequence in its endogenous locus. The construct was transformed into mouse embryonic stem cells to generate corrected stem cells, and the said stem cells were injected into blastocysts to generate chimeric mice. The chimeric mice were crossed with C57/B6J to obtain germ-line transmission as described in (Li et al., 2011). All experimental mice were backcrossed for at least 9 generations with C57/B6J.

### 2.2. Animal housing and genotyping

All the experiments were performed using male mice. *Mecp2*^*S80A;S421E/y*^ mice have been backcrossed to C57BL/6 background for more than ten generations. Mice were housed in 12 h light (07,00–19:00)/12 h dark (19,00–07,00) cycle. Mice were housed with no more than 5 animals per cage. All protocols were approved by the Institutional Animal Care and Use Committee at University of Wisconsin–Madison.

The genotype of the animals was determined using GoTaq Green Master Mix (Promega, M7123). All PCR reactions use the protocol with: 3 min hot start at 94 ^◦^C, 40s of denaturation at 94 ^◦^C, 40s of annealing at 56 ^◦^C, 40s of extension at 72 ^◦^C, repeated 35 times followed by a final extension at 72 ^◦^C for 2 min. Primers for genotyping include: NcoI-F, 5'-TCACAAGTTAACAGTGCCAGCTGC-3′; NcoI-R, 5'-ACCCCTTGGGACTGAAGTTACAGA-3′; FLAG-F, 5'-CTTGTCGTCGTCGTCCTTGTAG-3′; FLAG-R, 5'-AGCCTCAGGACTTGAGCAGCA-3′.

All breeding animals and majority of the experimental animals were sequenced at the S80 and S421 sites to validate point mutation. The PCR protocol is the same as previously described. Primers for sequencing are as below: S80-F, 5'-ATGAGCCACTACAACCTTC-3′; S80-R, 5'-AGACCTAATCCACCACCAA-3′; S421-F, 5'-TAAGAAGGAGCACATCATC-3′; S421-R, 5'-CCATAAGGAGAAGAGACAACA-3′.

### 2.3. Protein extraction and western blot

Mouse brain tissues were dissected on ice, homogenized, and lysed in RIPA buffer supplemented with protease inhibitor cocktails (Roche) and PhosSTOP phosphatase inhibitor (Roche). Total protein was extracted, and the protein concentration was measured with Coomassie Brilliant Blue staining and spectrophotometer.

For western blot assay, the protein extracts were loaded with SDS sample buffer and size-separated with 4% and 12% SDS/PAGE gel following general protocol. GAPDH (Santa Cruz, sc-32233, 1:2000) and MeCP2 (Abcam, ab2828, 1:1000) were used for the primary antibody. Secondary goat anti-mouse or anti-rabbit IgG-horseradish antibodies (CalBiochem) were used, and detection was performed using Odyssey (LICORbio) and the protein levels were quantified with Image Studio 5.0 (LICORbio).

### 2.4. Immunofluorescent staining and imaging

Mouse brains were collected after transcardial perfusion, sucrose cryoprotection, and fixed with 4% PFA. Cryosections of 40 um thickness were collected followed by standard immunofluorescence staining protocol. The brain sections were first blocked with 10% normal donkey serum and permeabilized with 1% Triton-X100 (Millipore Sigma, WXBF440V). DAPI (1:1000) and MeCP2 (Cell Signaling Technology, D4F3, 1:500) were used followed by secondary Alexafluor 488 conjugated donkey-anti-rat igG (1:2000, Invitrogen) probing. Finally, the tissues were mounted on a glass slide and coat with Fluoromount-G (SouthernBiotech, 0100–01) and let air dry overnight.

The fluorescent images were captured using Leica Stellaris 8 Confocal.

### 2.5. Behavioral testing

*Mecp2* A;E and WT male littermates aged between 8 and 15 weeks old were used for behavioral tests. The test sequence was conducted following the order of stress from low to high. Mice were moved to the test room or the holding cages and let them habituate for at least 30 min prior to the test. All testing arenas and objects were carefully cleaned and deodorized before and after each test. Animals were allowed 2–3 days interval between two assays. Testing videos were analyzed by ANY-Maze (Stoelting). 24 WT and 25 A;E male littermates were used for the running wheel, open field, sociability, accelerating rotarod, and fear conditioning tests.

#### 2.5.1. Running wheel test

Each animal was singly housed with a running wheel in the cage. Animals were allowed 1 day of habituation after being transferred into the experiment cages. The recording started the start of the dark cycle (6 PM – 6 AM) and track the wheel running for 24 h.

#### 2.5.2. Open field test

Each animal was individually placed at the center of the testing arena (41 × 41 × 30 cm) and let freely explore for 30 min. Their movements were detected and recorded by laser beam and detectors located on the sides of the arena (Omnitech Electronics Inc., USA). The travel distance and the time spent in the center and periphery of arena were measured by the tracking system.

#### 2.5.3. Sociability test

The sociability test was performed in a 3-chambered arena (each chamber: 40cm long × 22cm wide, and 23cm high walls), with a small door enabling access into each chamber. A wire cage is placed in the left and right chambers. Each animal was first allowed to explore the arena for 10 min (habituation session), and the wire cages are empty during this stage. In the second stage (social stage), a stranger mouse (stranger 1, 6–8 weeks old wildtype male) was placed in one of the wire cages whereas the wire cage in the opposite side remains empty. The test mouse was allowed to freely explore and interact with the stranger 1 for 10 min. In the last stage (novel session), a novel unfamiliar mouse (stranger 2, 6–8 weeks old wildtype male) was added to the previously empty wire cage on the opposite side to the stranger 1. The test mouse was tested for 10 min for evaluation of the social novelty recognition test. All experiments were videotaped and scored by ANY-maze (Stoelting). The discrimination index was calculated as the difference between the percentages of time spent exploring the stranger 1 and empty cage in social phase, or stranger 2 and familiar stranger 1 in novel phase.

#### 2.5.4. Accelerating rotarod test

Each mouse was placed on the lane of the rotarod. Once the mouse is stable, the rod starts running at 4 rpm and uniformly accelerates to 40 rpm over a 5-min period (Rota-Rod 2). The time latency of each mouse falling off the rod is recorded. Test animal is allowed 30 min interval between each trail. A total of 4 trails were recorded for each animal.

#### 2.5.5. Fear conditioning test

The fear conditioning test was controlled and recorded by ANY-maze (Stoelting).

24 h before the training, the mouse was first allowed to habituate to the chamber for 6 min with no sound or electro stimuli. During the training stage, the mouse was placed in the chamber and let explore for 2 min. Then, a white noise (87 dB) was sounded for 30s followed by a mild electrical foot-shock (0.5 mA, 1.5 s). The same noise-shock stimulus was repeated one more time for a total of 2 stimuli after a 2 min interval. The animal was then returned to the home cage. The context test was performed 22 h after the training. During the context test, the animal was placed in the same training chamber for 5 min and then returned to the home cage. The cue test was performed 2 h after the context test. Colored boards were placed on the sides and the floor of the training chamber, and 70% isopropanol instead of 70% ethanol was used for cleaning to change the “context” of the chamber. The animal was placed in the chamber and let explore for 2 min. Then, the same white noise (87 dB, 30s) was sounded but no electrical foot-shock was given. The sound was played again after a 2 min interval for a total of 2 times. The animal was then returned to the home cage.

#### 2.5.6. Elevated plus maze test

Each testing animal was placed at the center part of the elevated plus maze. The animals were allowed to freely explore the arena for 15 min. The traces of the animals were tracked by ANY-maze (Stoelting) and the ratio of time spent in the open arms to that spent in the closed arms was calculated.

#### 2.5.7. Light-dark chamber test

Each testing animal was placed in the dark chamber at the start of the test. The animals were allowed to freely explore both light and dark chambers for 10 min. The appearance of and the moving trace in the light chamber were recorded from a top camera and analyzed by ANY-maze (Stoelting). The ratio of time spent in the light chamber to that spent in the dark chamber was calculated.

#### 2.5.8. Statistics

For the running wheel, open field, three-chamber sociability, elevated plus maze, and light–dark chamber assays, animals with a total travel distance below the threshold of [mean – 1.5 × SD] were classified as inactive and excluded from subsequent analyses. Exclusion thresholds were calculated separately for WT and A;E groups. The number of animals meeting the inclusion criteria for each assay and the detailed statistics results are provided in Table S1.

Data normality was assessed using the Shapiro–Wilk test, and homogeneity of variance was evaluated using Levene's test. For comparisons between two groups, a two-tailed Studenťs *t*-test was used when data were normally distributed and variances were equal. When data were normally distributed but variances were unequal, a two-tailed Welch's t-test was applied. For datasets that did not satisfy the assumption of normality, a two-tailed Mann–Whitney *U* test was used. Statistical significance was defined as *p* < 0.05.

For the fear conditioning, 3-chamber sociability, and accelerating rotarod assays, repeated-measures ANOVA were performed with genotype and the testing phases as the between-subject factor, followed by post-hoc test using independent t-test with Bonferroni correction. Statistical significance was defined as p < 0.05.

Post hoc power analysis was performed following the initial behavioral assessment to estimate the effect size and the number of animals required to achieve 80% statistical power at a significance level of 0.05.

### 2.6. Electrophysiology

Coronal brain slices were prepared from adult WT or AE mice as described. In summary, mice were euthanized with CO2, and the brains were isolated. Using a microtome (VT1200S, Leica Microsystems), coronal brain slices (300 μm) were prepared in ice-cold modified artificial cerebrospinal fluid (aCSF) (in mM: 93 NMDG-Cl, 2.5 KCl, 1.2 NaH2PO4, 30 NaHCO3, 20 HEPES, 25 glucose, 5 sodium ascorbate, 2 Thiourea, 3 sodium pyruvate, 10 MgSO4, and 0.5 mM CaCl2, pH = 7.35) bubbled with 95%O2/5%CO2. Then the slices were incubated in normal aCSF (in mM: 124 NaCl, 2.5 KCl, 2.5 CaCl2, 1.2 MgCl2, 1.25 NaH2PO4, 26 NaHCO3, and 15 glucose) at room temperature for at least 1 h. Slices were then transferred to a recording chamber perfused with normal aCSF. Cells were visualized using an Olympus BX51WI microscope equipped with infrared differential interference contrast (IR-DIC) optics. Patch pipettes were pulled from thick-walled, filamented borosilicate glass (Sutter Instruments, BF150–86–10) using a Sutter Micropipette Puller (P-1000; Sutter Instruments) to a tip resistance of 5–8 MΩ. Whole-cell patch-clamp recordings were made using a MultiClamp 700B amplifier, digitized with a Digidata 1440 A, and acquired with pCLAMP 10 software (Molecular Devices, USA).

To be included in the analysis, a cell was required to have a seal resistance greater than 1 GΩ before breaking into whole-cell mode and an initial access resistance <20 MΩ and < 10% of the input resistance (Rin).

For recordings of intrinsic properties and spontaneous excitatory postsynaptic currents (sEPSCs), the internal solution contained 140 mM K-gluconate, 7.5 mM KCl, 10 mM HEPES-K, 0.5 mM EGTA-K, 5 mM Mg-ATP, and 0.3 mM Li-GTP (pH 7.2, adjusted with KOH). After the establishment of a stable whole-cell configuration, the resting membrane potential (RMP) of each cell was recorded. Each cell was then subjected to a standardized stimulus protocol, including both voltage- and current-clamp paradigms, to extract features for cross-cell comparisons. Subthreshold membrane properties, including input resistance, membrane capacitance, membrane time constant, and sag ratio, as well as action potential features, including rheobase current, spike peak, spike threshold, full width at half maximum (FWHM), and firing frequency, were extracted using the IPFX Python package.

sEPSCs were recorded in voltage-clamp mode at a holding potential of − 70 mV. Events were analyzed using Python. The detection threshold was typically set at 5 pA and adjusted according to baseline noise. Recordings with unstable baselines were excluded from analysis. The frequency and amplitude of sEPSCs were measured.

Outlier detection was performed independently for each measured parameter using the 1.5× interquartile range (IQR) rule. Data points falling below Q1–1.5 × IQR or above Q3 + 1.5 × IQR were defined as outliers and excluded from subsequent statistical analysis.

### 2.7. Single cell libraries construction and data analysis

#### 2.7.1. Single cell nuclei suspension preparation for snMultiome

Mice were euthanized and the brains were carefully dissected in ice-cold PBS. Different brain regions were separated and moved to −80 ^◦^C for long-term storage until ready to use. For nuclei isolation, the frozen hippocampus was resuspended in 500ul of lysis buffer (10 mM Tris-HCl, 10 mM NaCl, 3 mM MgCl2, 0.10% Tween-20, 0.10% NP-40, 0.01% Digitonin, 1% BSA, 1 mM DTT, 1 U/uL RNase inhibitor) in a 1 ml douncer. The tissue was homogenized using a loose pestle and a tight pestle (30× each) without introducing air bubbles. The homogenized tissue suspension was incubated on ice for 15 min with a gentle pipette mixing in the middle. 500ul of chilled wash buffer (10 mM Tris-HCl, 10 mM NaCl, 3 mM MgCl2, 1% BSA, 0.10% Tween-20, 1 mM DTT, 1 U/uL RNase inhibitor) was then added to the suspension followed by gentle pipetting mix and then went pass through a 40-μm nylon cell strainer. 1000ul of Opti-Prep (Sigma, I8896) was added to the suspension and then centrifuge at 500 rcf for 10 min. Supernatant was carefully removed without disturbing the nuclei pellet. The pellet was washed with 1 ml chilled wash buffer following by centrifuging at 500 rcf for 5 min at 4 ^◦^C. Perform nuclei count after the first wash. Finally, remove supernatant and resuspend it in chilled diluted nuclei buffer. Perform a second nuclei count to ensure quantification accuracy.

#### 2.7.2. Library construction and sequencing

In this study, the aim sample size for each sample was a target recovery of 10,000 nuclei per capture. The sequencing libraries were constructed following the Chromium Next GEM Single Cell Multiome ATAC + Gene Expression user guide (CG000338 Rev. F). The Chromium Next GEM Single Cell Multiome ATAC + Gene Expression Reagent Bundle (PN-1000283) were employed. The GEM generation process was accomplished with a Chromium Controller (10× Genomics) and Chromium Next GEM Chip J Single Cell Kit (PN-1000234). The libraries were indexed utilizing Single Index Kit N Set A (PN-1000212, for ATAC) and Dual Index Kit TT Set A (PN-1000215, for cDNA). Sequencing was performed on the novaseq x plus platform (Illumina) for a targeted depth of 30,000 reads per nucleus.

#### 2.7.3. 10× snMultiome data alignment

The 10× Multiome dataset (Gene Expression + ATAC) was processed using the Cell Ranger ARC pipeline (v2.0.0). The data was aligned using the GRCm39 (GCF_000001635.27) as the reference assembly and performed a quality check on the output of the Cell Ranger ARC pipeline for downstream analyses.

#### 2.7.4. snRNA-seq data processing

The Seurat (v5.3.0) R package (Hao et al., 2024) was used to pre-process and analyze the snRNA-seq data after alignment. First, the SoupX (v1.6.2) (Young and Behjati, 2020) was utilized to remove the ambient RNA contamination. Then, low-quality nuclei (a nucleus that has a number of features less than 1000, UMI count less than 10% quantile or greater than 90% quantile, and mitochondrial content greater than 5%) were removed from the downstream analysis. Finally, the DoubletFinder (v2.0.3) R package (McGinnis et al., 2019) was used to filter out doublets in each sample separately. The cleaned dataset was processed following the default Seurat pipeline. The SoupX-corrected counts were normalized using NormalizeData() function with a scale of 10,000. The top 2000 highly variable genes were obtained using the FindVariableFeatures() function in Seurat. The highly variable genes were then sealed and used to estimate principal components with the RunPCA() function. The dimensionality of the data was inquired using an elbow plot, and the top 30 principal components were used for nonlinear dimensional reduction using Uniform Manifold Approximation and Projection (UMAP) method. The batch correction across our samples was performed using the Canonical Correlation Analysis (CCA) based approach implemented in Seurat.

#### 2.7.5. Cell clustering, cell type annotations, and differentially expressed genes (DEGs) calling

Transcriptomically similar cells were clustered together using the FindCluster() function in Seurat with the top 30 principal components. The cluster resolutions of 1, 1.5, and 2.0 (yielding 42, 52, and 56 clusters, respectively) were tested and the cluster resolution of 1.5 was taken as the optimum resolution. Nuclei were clustered into seven major classes based on the expression of following marker genes: excitatory pyramidal neurons (IT-ET Glut – *Slc17a7*), granule neurons (DG-IMN Glut – *Slc17a7*, *Prox1*), inhibitory neurons (GABA – *Gad1*), astrocytes (Astro – *Aqp4*), oligodendrocytes (Oligo – *Sox10*, *Mobp*), oligodendrocyte progenitor cells (OPC – *Sox10*, *Mobp*, *Pdgfra*), and microglia cells (MG – *Tmem119*). Differentially expressed genes were called for each cell type between WT and A;E using FindMarkers() function under the default settings (test.use = wilcox, min.pct = 0.01, min.log_2_FC = 0.1). DEGs were called using the threshold of adj.*p* < 0.001.

#### 2.7.6. Gene ontology (GO) enrichment analysis

The GO analysis was performed using g:Profiler version e114_eg62_p19_27110d83 (Kolberg et al., 2023), with “*Mus musculus*” as the selected species. Enriched GO terms for cell-type specific top regulons favoring each genotype were called separately. For GO analysis, we focused on “GO Biological Processes” and subsequently focused on adjusted *P* value less than 0.05 for additional analysis.

#### 2.7.7. Evolution of the clustering stability and cell type separability

The accuracy of the cell type annotation depends on the ability of the clustering method to successfully group cells based on their transcriptomic similarity. Since the clustering method primarily uses a shared neighbor graph obtained at reduced dimensions, the cluster stability of the accuracy of the cell type annotations can be determined by investigating their cell neighbors. Here, we adopted a neighbor voting-based approach as described in (Gao et al., 2025; Ma et al., 2022; Risgaard et al., 2025). First, the original dataset was subsampled by randomly isolating cells without replacement. Here, we performed this analysis 3 times by isolating 10%, 25%, and 50% of the total cells for each simulation. The subsampled dataset was preprocessed following the standard preprocessing pipeline that includes log-normalization, scaling, and obtaining principal components. Next, the 20 nearest neighbors for each cell were identified using the reduced-dimensional space, and new cell type predictions were made using a neighbor voting approach, where each cell was labeled based on the most abundant cell type among its neighbors (requiring at least 60% agreement). Then, we computed the area under the receiver operating characteristic (AUROC) values for each cell type using a subset of 2500 randomly selected cells, comparing the original and predicted annotations. The AUROC values are depicted in Fig. S2c.

#### 2.7.8. Gene regulatory network inference using snRNA-seq

Cell type–specific gene regulatory networks (GRNs) for DG-IMN and IT-ET glutamatergic subclasses were inferred using the SCENIC (Aibar et al., 2017) framework implemented in pySCENIC (Van de Sande et al., 2020) (v0.12.1 + 8.gd2309fe). To reduce the noise inherent to snRNA-seq data due to dropout events, pseudo-bulk cell profiles were generated. To preserve both cellular identity and genotype information, the following steps were performed after isolating cells corresponding to a given cell type and genotype:1) Isolation of cells for a given cell type and genotype; 2) Principal component analysis followed by Harmony integration (Korsunsky et al., 2019) to remove batch effects; 3) Identification of the 10 nearest neighbors for each cell using k-nearest neighbors based on batch-corrected Harmony components; 4) Random selection of 10% of the cells as root cells for pseudo-bulk profile generation; and 5) Aggregation of gene expression profiles from the 10 closest neighboring cells for each pseudo-bulk root cell.

The resulting pseudo-bulk cells (2637 IT-ET cells with 999 from A,E and 1638 from WT, 1304 DG-IMN cells with 484 from A,E and 820 from WT) were used for downstream GRN inference. Only the protein-coding genes were incorporated in the GRN inference. Log-normalized gene expression data were used to infer a co-expression network by linking transcription factors (TFs) to putative target genes (TGs) based on gene–gene correlations using GRNBoost2 (Moerman et al., 2019). Mouse TF lists provided in the pySCENIC package (https://resources.aertslab.org/cistarget/tf_lists/) were employed. Only the 50% of the top TF-TG combinations were kept for the second step of the SCENIC pipeline in order to reduce false positives. The resulting putative target genes were further refined by assessing enrichment of TF-binding motifs in the upstream regulatory regions using RcisTarget and the *Mus musculus* (mm10) databases provided with pySCENIC (https://resources.aertslab.org/cistarget/databases/, https://resources.aertslab.org/cistarget/motif2tf/). This approach resulted in a directed gene regulatory network linking TFs to their predicted target genes.

Notably, the inferred GRNs represent consensus TF–target gene relationships derived from cells of both genotypes, thereby integrating information across phenotypes. Genotype-specific regulatory behavior was assessed by estimating regulon activities. Regulons were defined as gene sets comprising all target genes associated with a given TF. TFs with fewer than five target genes were excluded from further analysis.

Regulon activity was quantified using the UCell (v2.12.0) (Andreatta and Carmona, 2021) algorithm, which computes per-cell activity scores for gene sets, with higher UCell scores indicating higher regulon activity. To filter out low-activity regulons, a cutoff of 25 was applied to the total regulon activity. Genotype specificity of regulon activity was evaluated using a two-sided Wilcoxon rank-sum test, along with Cliff's *δ* effect size (effsize v0.8.1). The 95% confidence intervals for Cliff's *δ* were estimated by bootstrapping with 1000 iterations. Positive Cliff's delta values indicate specificity towards the “A;E” genotype, whereas negative values indicate specificity towards “WT”.

Regulons were categorized into four impact categories based on effect size: (1) no impact (|δ| <0.147), (2) small effect (0.147 ≤ |δ| <0.3), (3) medium effect (0.3 ≤ |δ| < 0.474), and (4) large effect (|δ| ≥ 0.474) (Meissel and Yao, 2024). The resulting gene regulatory networks and regulon analysis results are provided in Supplementary Table S3.

#### 2.7.9. snATAC-seq data processing

The Signac (Stuart et al., 2021) (v1.14.0) and Seurat (Hao et al., 2024) (v5.2.0) packages were used for processing snATAC-seq data. Since our data are paired multiomic data, that measured both modalities on the same nuclei, we first isolated the nuclei that passed the snRNA-seq quality control metrics. This enabled us to assign cell type identities a priori. Accurate chromatin accessibility analysis relies on precise peak calling, as peaks called across the entire cell population may overlook those unique to smaller subpopulations (Stuart et al., 2019). Thus, peak calling was performed using the MACS2 (Zhang et al., 2008) method implemented within Signac by grouping cells based on cell type and genotype. Then the inferred peak sets were combined using the reduce() function in Signac to obtain a consensus peak set. Then the peak count matrix was generated using the fragment files, and per-cell quality control metrics were estimated. This includes transcriptional start site (TSS) enrichment, nucleosome signal, and the number of peaks. The low-quality nuclei were identified and filtered based on whether the per-cell peak count was less than 1000 or greater than 100,000, nucleosome signal greater than 4, and TSS enrichment less than 2. The retained data were then normalized using the term frequency inverse-document frequency (TF-IDF) method (Stuart et al., 2019) in Signac.

#### 2.7.10. Differential accessibility peaks

Differentially accessible peaks (DAPs) were identified using the Seurat (v5.3.0) and Signac (v1.14.0) framework. Peak features were prefiltered to retain only canonical mouse chromosomes (chr1–chr19, chrX, chrY). For each of the seven major cell types (Astro, ExN, Granule, InN, MG, Oligo, and OPC), DA peaks between A;E and WT nuclei were computed with the FindMarkers() function in Signac, using the logistic regression test (test.use = “LR”) and requiring a peak to be detected in at least 10% of cells in either group (min.pct = 0.1). The average log fold-change was defined as log_2_(A;E / WT), and we applied the default log_2_ fold-change threshold (logfc.threshold = 0.1). This corresponds to a minimum effect size of ≈1.07-fold difference in accessibility between A; E and WT (since 2^0.1 ≈ 1.07). Peaks were considered significant if they passed both the log_2_ fold-change threshold and an adjusted *p*-value <0.05. The resulting DA peak sets were then exported and used for downstream genomic annotation and peak-to-gene linkage analyses.

#### 2.7.11. ATAC peak annotations

To annotate cell-type–specific accessible chromatin regions, we focused on differentially accessible (DA) peaks identified from logistic regression testing (adjusted *p* < 0.05) using the Seurat (v5.3.0) and Signac (v1.14.0) framework. For each cell type, we selected DA peaks for annotation using ChIPseeker (v1.44.0) with gene annotations from TxDb.Mmusculus.UCSC.mm39.knownGene (mouse mm39 genome) and org.Mm.eg.db. Peaks were converted to GRanges objects using their genomic coordinates and annotated using annotatePeak() with a transcription start site (TSS) window of − 3 kb to +3 kb. Each peak was assigned a single annotation based on the default priority. The annotated peak categories for each cell type's DA peaks were then summarized and visualized as pie charts. These charts display the proportion of DA peaks falling into each category (promoter, UTRs, exonic, intronic, etc.) for that cell type, highlighting differences in the genomic distribution of accessible chromatin features among cell types.

#### 2.7.12. Inferring cell-type peak-to-gene links

For each cell-type, we applied Signac's LinkPeaks() function to compute peak-to-gene correlations with identified DEGs as described previously. A 100 kb window around each DEG's transcription start site was used to identify nearby peaks, and Pearson correlation coefficients were calculated between each peak's accessibility and the gene's expression across single cells. Peak-to-gene linkages were derived both using all cells in each cluster and separately using only cells of each genotype (A;E vs WT) to capture genotype-specific interactions. Resulting peak–gene pairs were filtered to retain those with an absolute Pearson correlation ≥0.05 and an adjusted p-value <0.05 as significant linkages.

#### 2.7.13. Motif scanning

The motif scanning is performed using Motif Alignment & Search Tool (MAST) (Bailey and Gribskov, 1998).

### 2.8. MERFISH sample preparation and data analysis

#### 2.8.1. MERFISH sample collection and preparation

Mice (wildtype and A;E, 9-week-old male littermates) were euthanized and the brains were quickly collected and embedded in a square tissue mode with OCT (Tissue-Tek, 4583) mounting medium. The brains were flash frozen in dry ice-chilled isopentane and stored at −80^◦^C until sectioning.

To prepare cryosections for MERFISH, a 10-μm-thick coronal slice containing target regions was mounted onto the center of a coverslip (Vizgen, 10500001), fixed in 4% paraformaldehyde in PBS in a 6cm petri dish, washed with PBS, and permeabilized and stored in 70% ethanol at 4^◦^C until MERFISH probe hybridization. MERFISH was performed according to Vizgen's protocol Fresh and Fix Frozen Tissue Sample Preparation (91,600,002 Rev. E). MERSCOPE sample prep kit (Vizgen, 10400012) was employed. Then, the tissue section was stained with DAPI-poly(T) and imaged following the MERSCOPE Instrument User Guide (91,600,001 Rev. G). Once MERFISH imaging process was completed, the output files were transferred for in-depth analysis with MERFISH Visualizer and our customized bioinformatic pipeline.

The gene panel was constructed based on the commercially available 300-gene set from Vizgen (BP1587). The MERFISH gene panel contains cell-type markers, high fold-change DEGs identified from prior 10× experiment which is biased towards neurons, and genes known to be dysregulated in Mecp2-KO animal.

#### 2.8.2. MERFISH imaging

The MERFISH imaging was performed following the MERSCOPE Instrument User Guide 91,600,001 Rev. G. The imaging process was controlled by the Vizgen MERSCOPE program with the default settings (both polyT and DAPI channels “on”, scan thickness: 10 μm). The imaging occurred in 3 batches with 2 WT and 2 A;E hippocampi in each batch.

#### 2.8.3. Processing of MERFISH dataset

The transcripts were decoded and error-corrected correction via the MERSCOPE software pipeline, individual cells were segmented using the machine learning model cellpose2, which was custom trained on DAPI-poly(T) stained slices collected previously, captured at the same resolution. Transcripts were assigned to individual cells. Next, the hippocampal regions are manually selected via MERSCOPE Visualizer for downstream analysis. The data was processed using Seurat (v5.3.0) R package. Low-quality cells (a cell that has a transcript count of less than 50 or greater than 6000, and cell volume less than 200 or greater than 3 times of the medium) were removed from the downstream analysis. The DoubletFinder (v2.0.3) R package was used to filter out doublets in each sample. Then, the data went through normalization and variance stabilization with SCTransform() function, and principal components estimation with the RunPCA() function. The dimensionality of the data was identified using an elbow plot, and the top 20 principal components were used for nonlinear dimensional reduction using the RunUMAP() function. Different batches were integrated using harmony-based integration. The cells were clustered based on transcriptome similarity using the FindClusters() function in Seurat with the top 20 principal components. The cluster resolutions of 1,1.5. and 2 (with 26, 33, and 36 clusters, respectively) were tested and the cluster resolution of 1.5 was taken as the optimum resolution. The cell types were annotated with the same set of markers as used for snRNA-seq analysis. Cell type annotations were refined and corrected using spatial coordinates. IT-ET Glut cells were further subclustered using the FindSubCluster() function with resolution of 1.

#### 2.8.4. Filtering of differentially expressed genes in MERFISH dataset

The differentially expressed genes between the two genotypes were called using studenťs *t-*test and Bonferroni correction was applied for population-adjusted *p* value. Low expressors (transcripts with counts of less than twice the mean value of the 15 blank reads) were removed from the analysis. Log fold changes were computed using a base of 2.

#### 2.8.5. Cell-cell interaction analysis of the 10× multiome data

For each genotype group, the cell type interactions (CCIs) were inferred using LIANA (Dimitrov et al., 2022), which uses a combination of 16 resources and 7 CCI methods to find consensus interactions. A threshold of 0.01 was used for both magnitude rank and specificity rank to extract significant CCIs. Since the resources used by LIANA contain manually curated ligand-receptors not specific to the brain, we also ran NeuronChat (Zhao et al., 2023), which mainly focuses on neuronal ligand-receptor interactions.

#### 2.8.6. *Inferring 10*× *spatial coordinates using Tangram and region-specific DA analysis*

Spatial coordinates for individual 10× nuclei were inferred using Tangram (v1.0.4) (Biancalani et al., 2021), which aligns single-cell transcriptomic profiles to spatial transcriptomic reference data. The preprocessed MERFISH dataset served as the spatial reference, and the IT-ET Glut nuclei from the 10× snMultiome dataset were used as the query. Tangram was trained in gene-mode using the set of shared genes between the two modalities and run with GPU acceleration to optimize mapping probabilities. The mapping matrix was normalized so that each 10× nucleus had a probability distribution summing to one across all MERFISH cells. For each 10× cell, the argmax probability was used to assign its most likely spatial position, resulting in predicted x,y coordinates. DEGs and DAPs from the four subregions (CA1, CA2, CA3, and Mossy) were called as previously described.

## 3. Results

### 3.1. Mecp2 ^S80A;S421E^ mice show decreased mobility, increased central area exploration, and increased contextual memory

To test the in vivo function of *Mecp2 S80A;S421E* mutation, we generated *Mecp2 S80A;S421E* (A;E) knock-in mouse line that carries mutation of serine 80 to alanine to abolish phosphorylation, and serine 421 to glutamic acid to mimic phosphorylation (Fig. 1A). To confirm the knockin mutation, we amplified and sequenced a 400 bp length DNA fragment, with 200 bp flanking the S80 and S421 loci to ascertain the mutation status (Fig. 1B,C). No obvious RTT-related phenotype was observed in the A;E mice through the development. Western blot of lysates from different brain sub-regions showed that the total MeCP2 protein level in the A;E mutant is unchanged compared with the wild type (WT) (Fig. 1D, E), suggesting that total MeCP2 expression levels in the brain are not affected by the double phosphor mutation. Notably, the A;E band is slightly higher than the wild-type band, which is consistent with the expected mobility shift due to the mutation to glutamic acid at the 421 location. No obvious difference was observed in the immunostaining of MeCP2 expression levels throughout the brain, including granule neurons in the hippocampal DG area (Fig. 1F).

Previous work have shown that MeCP2 is an intrinsically disordered protein, with most of its polypeptide chain lacking a fixed three-dimensional structure (Quintanilla-Sierra et al., 2019). Both S80 and S421 are located within these disordered regions, making traditional structure-function analysis challenging. Rather than attempting to decipher phosphorylation-induced conformational changes, we focused on exploring functional consequences.

To characterize the functional impact of the A;E mutation at the animal level, we performed a battery of behavior assays to evaluate the phenotypical changes in the adult A;E mice (Fig. 2A). Only male littermates were used to avoid mosaic expression of MeCP2 caused by random X chromosome inactivation. Given that *Mecp2* modified mouse models exhibit delayed phenotype onset compared to human cases, all examinations focused on early adulthood to ensure detectable phenotypes and comparability with published work.

The behavioral assays aimed to evaluate various functions that are reported to be affected in previously reported *Mecp2* mutant mouse models (Guy et al., 2001; Li and Chang, 2014). The sequence of behavioral assays was arranged in ascending order of stress intensity to minimize the impact of preceding tests on the animals. Each assay was separated for 2–3 days to ensure full recovery of tested mice (Fig. 2A).

The first assay is the 24-h running wheel test (RW), in which the A;E mice exhibited significantly decreased dark cycle running activity (Fig. 2B, *p* = 0.05, student *t*-test).

To further confirm the locomotion phenotype and to test the anxiety levels, we next performed the open field (OF) assay. Specifically, the total moving distance during the 30-min testing session decreased significantly (Fig. 2C, *p* = 3.177 × 10^−5^, student t-test). We also noticed that the vertical movement counts during the test session are lower in the A;E mice compared to the counts in the wildtype littermate controls (Fig. 2D, p = 0.006, student t-test), suggesting a decrease in the action of standing on their hindlegs, indicating the locomotion defect may be due to reduced hindleg strength. Furthermore, heatmaps show that the A;E mice spent more time in the central area compared to the wild-type controls (Fig. 2E), and there is significantly longer exploration duration and distance in the central area of A;E mice compared to their wild type littermates (Fig. 2F, p = 0.032, student *t*-test; Fig. S1A, *p* = 0.025, student t-test). However, elevated plus maze and light-dark box experiment performed on a separate and smaller cohort of mice detected no difference between A;E and the control (Fig.S1B-C), suggesting that the increased central area exploration in the open field may not be due to reduced anxiety. An alternative explanation is altered spatial cognition in the A;E mutant compared to the wildtype. Collectively, results from the open field assay demonstrated locomotor deficits accompanied by increased central area exploration in the A;E mutant.

Finally, a fear conditioning (FC) assay was performed to test the fear memory of the A;E mutant. The A;E mice showed significantly increased freezing time during the context test compared to their wild-type littermates (Fig. 2G, p = 9.61 × 10^−5^, repeated measures ANOVA), whereas the freezing time during the cued test is unchanged. Due to the differential contribution of amygdala and hippocampus to fear conditioning assay (Maren, 2008), where contextual tests are often thought to depend on both amygdala and hippocampus while cued test only depends on the amygdala, our results suggest that there may be a difference in hippocampal function between A;E and wild-type mice.

The A;E mice showed no change in the 3-chamber sociability test or the accelerating rotarod, suggesting no functional difference in sociability or motor learning (Fig.S1D, S1E). Statistical details for all behavioral data can be found in Table S1.

### 3.2. Electrophysiological analysis reveals enhanced spontaneous excitatory postsynaptic currents (sEPSCs) in Mecp2 ^S80A;S421E^ hippocampal CA1 pyramidal neurons

To look for associated electrophysiological changes, we performed whole-cell patch-clamp recording of CA1 pyramidal neurons in acute hippocampal slices prepared from adult A;E mice and their wild type littermates. A comprehensive list of subthreshold membrane properties, including resting membrane potential (RMP), input resistance (Rin), membrane capacitance, membrane time constant, and sag ratio; as well as evoked action potential features, including rheobase current, spike peak, spike threshold, full width at half maximum (FWHM), and firing frequency were measured. No statistically significant difference in any of the above listed features was detected between A;E and WT (selected features such as RMP, Rin, rheobase, and spike peak are shown in Fig. 3C-F). Interestingly, when sEPSCs were recorded, we observed significant increase in both the frequency and amplitude of the sEPSCs in A;E when compared with the WT (Fig. 3A, p = 0.021; Fig. 3B, p = 0.011. Welch t-test), suggesting elevated presynaptic activities and enhance postsynaptic response. These electrophysiological changes are consistent with the observed behavioral phenotypes.

### 3.3. Single-nuclei gene expression profile reveals glutamatergic-neuronal-oriented transcriptional changes in Mecp2 ^S80A;S421E^ hippocampus

Since the fear conditioning assay highlighted hippocampus as a brain region with altered function, we decided to focus on this region and performed an unbiased single nuclei multiome sequencing (snMultiome) to detect the primary effects of A;E mutation on gene expression profile and chromatin state at the single cell level. Hippocampi from 3 A;E and 3 WT 9-week-old male littermate animals were sampled and snMultiome libraries were constructed. After stringent quality control and filtering, 60,889 nuclei were included for further analysis (WT: 36,057, A;E: 24,832), with no significant difference in the number of unique genes per cell, the number of transcripts per cell, the percentage of mitochondrial transcripts per cell, or the percentage of ribosomal transcript per cell among individual samples (Fig.S2A).

After batch effect removal, cells were clustered and annotated into 7 major cell types based on the cell type marker genes expression levels (Fig. S2C): intratelencephalic-projecting and extratelencephalic-projecting glutamatergic neurons (IT-ET Glut), Dentate gyrus-immature glutamatergic neurons (DG-IMN Glut), GABAergic inhibitory neuron (GABA), astrocytes (Astro), oligodendrocyte (Oligo), oligodendrocyte precursor cells (OPC), and microglia (MG) (Fig. 4A). All major cell types are observed in both genotypes with no obvious change in the composition proportion (Fig. 4B), suggesting that the A;E mutation does not impact on hippocampal cell type composition. The clustering stability and cell type separability were carefully evaluated to ensure the accuracy of cell clustering and annotation (Fig. S2B). The number of unique genes per cell, the number of transcripts per cell, and the percentage of mitochondrial transcripts per cell were compared among various cell types and to published datasets (Franjic et al., 2022) to ensure the quality of each cell type is reasonable and comparable (Fig. S2D).

Next, differentially expressed genes (DEGs) between the two genotypes were called from each individual cell type (Fig. 4C and S3A-G, Table S2). DEGs were highly enriched in neurons, especially the glutamatergic excitatory neurons. 4761 DEGs were detected from the DG-IMN Glut cells, followed by 3496 DEGs in the IT-ET Glut and 1594 DEGs in the GABA. The non-neuron cells, in contrast, have much fewer DEGs detected compared with neurons, with 108 DEGs in Astro, 1052 in Oligo, 28 in OPC, and 54 in MG. This result is consistent with the prior knowledge that the phosphorylation states at S80 and S421 are driven by neuronal activities. It is highly likely that the transcriptional regulatory function of A;E mutation may be biased towards neurons, especially glutamatergic neuron populations. Furthermore, the magnitudes of transcriptional fold changes were generally mild in all cell types, with majority of log2FoldChange smaller than 2 (Fig. S3A-G). This pattern is similar to other published sequencing data on *Mecp2-null* tissue, where dysfunction of MeCP2 caused a broad yet modest impact to transcriptional profile (Boxer et al., 2020; Chahrour et al., 2008; Gulmez Karaca et al., 2018).

To better understand the regulatory effect from the A;E mutation and search for altered signaling pathways, we focused on the glutamatergic neuron cell categories (IT-ET Glut and DG-IMN Glut) and performed a gene regulatory network (GRN) analysis. Cell-type-specific consensus GRNs across genotypes were inferred linking transcription factor (TF) to target genes (TG) followed by regulon inference (i.e., regulatory subnetworks named after a TF and consisting of the TGs regulated by that TF). The regulon activities were then calculated using the UCell method (Andreatta and Carmona, 2021), in which a per-cell score is assigned to each cell based on the relative expression levels of the genes within each regulon. The differential enrichment of the regulons across genotypes were explored in terms of Wilcoxon rank sum test and the Cliff's delta, effect size calculations (Fig. 4D, F, Table S3).

In IT-ET Glut neurons, 27 regulons were identified as exhibiting significant changes in regulatory influence between WT and A;E, with 11 favoring WT and 16 favoring A;E (Fig. 4D). However, the five regulons with the largest absolute Cliff's delta values all favored WT, including *Nfe2l1, Usf2, Fos, JunB*, and *Egr1*. In DG-IMN Glut population, a total of 49 regulons exhibited significant changes in regulatory influence between WT and A;E, with 14 favoring WT and 35 favoring A;E (Fig. 4F). Similar to the observations in the IT-ET Glut cells, the five regulons with the largest absolute Cliff's delta values all favored WT, including *Nfe2l1*, *Klf13*, *Nfyb*, *Bhlhe22*, and *JunD*. Interestingly, several regulons canonically involved in hippocampal-dependent memory were found differentially enriched in the two Glut cell populations. In IT-ET Glut, *Fos*, *JunB*, *JunD*, and *Egr1* (He et al., 2002; Penke et al., 2014; Strekalova et al., 2003) were enriched in WT (Fig. 4D). In DG-IMN Glut, *Jun*, *Cebpb*, *Ybx1*, and *Ilf2* (Calella et al., 2007; Hayden et al., 2024; Sherrin et al., 2011; Yamashita et al., 2023)were enriched in WT, while *Foxp1* (Araujo et al., 2017; Bacon et al., 2015) favors A;E (Fig. 4F). The concentrated presence of these key TFs aligns with the observations from the earlier behavioral phenotyping results, suggesting consistency between the molecular and the functional levels.

Then, Gene Ontology (GO) enrichment analysis was performed separately on the regulons favoring WT and those favoring A;E. Regulons with top absolute Cliff's delta values were used for analysis. The terms differentially enriched in either WT or A;E were compiled and summarized. Among the GO terms favoring WT, those related to communications and responses were found enriched across multiple regulons, including “regulation of cell communication”, “intracellular signal transduction”, “regulation of signaling”, “response to stimulus”, “response to stress”, and “learning or memory” (Fig. 4E). In contrast, among the terms favoring A;E, enrichment was observed for categories involving “phosphorylation”, “metal ion transport”, “synapse organization”, “synapse assembly”, and “axon guidance”“ (Fig. 4G). Notably, within regulons favoring A;E, the gene subnetworks inferred in the DG-IMN Glut population exhibit a greater number of TG per TF compared to those in IT-ET Glut cells, leading to a higher count of enriched GO terms and more significant *p* value. In contrast, an opposite trend was observed in regulons favoring WT, where the gene subnetwork predicted in IT-ET Glut cells contain more members than those in DG-IMN Glut cells, again resulting in more GO terms and more pronounced p value in the IT-ET Glut population. This pattern suggests that the regulatory impact of A; E mutation is highly cell-type specific.

Finally, we attempted to identify altered cell-cell interactions (CCI) induced by the A;E mutation. A LIANA (Dimitrov et al., 2022) based framework supplemented with NeuronChat (Zhao et al., 2023) for neuronal signaling specialization pipeline was employed on a fraction of dataset. Stringent thresholds for both interaction magnitude (ranked by ligand-receptor expression levels) and specificity (ranked by cell type exclusivity) were applied to filter and retain high-confidence CCI results. A higher number of CCI pairs was identified in the A;E (18 pairs) compared to WT (14 pairs) (Table S4). Among the genotype-specific CCIs, 4 interaction pair was unique to WT, and 7 pairs were specific to A;E. Notably, CCIs that involve the participation of non-neuron populations exhibited higher weight compared to those involve only neurons (Fig.S3H), suggesting that non-neurons may play an important role despite modest transcriptional changes.

### 3.4. Chromatin accessibility signatures in Mecp2 ^S80A;S421E^ hippocampus congruent with the gene expression dataset

To gain a better understanding of different layers of regulatory elements, we integrated the snATAC-seq information which was simultaneously generated from the snMultiome assay with gene expression data. This multiome data allows us to inspect the regulatory impact of A; E mutation from multiple aspects.

After removing low-quality nuclei (peak count per cell less than 1000 or greater than 100,000, nucleosome signal greater than 4, and TSS enrichment score less than 2) (Fig.S4 A) and applying the cell type annotation results determined by gene expression profile, we were able to call ATAC-seq peaks from each cell type and genotype category and generate cell-type specific chromatin accessibility signatures. The UMAP clustering of cells based on integrated ATAC and gene expression (Fig. 5A) has similar cluster embedding patterns to the UMAP using gene expression data (Fig. 4A), suggesting a high consistency between the two datasets.

Next, differentially accessible peaks (DAPs) were called between two genotypes for each cell category. Similar to the gene expression dataset, DAPs were found to be highly enriched in glutamatergic neuron cell types, especially in IT-ET Glut (2589 DAPs). 497 DAPs were identified in GABA, followed by DG-IMN Glut (120 DAPs), Oligo (29 DAPs), Astro (22 DAPs), OPC (6 DAP), and MG (1 DAP) (Fig.S5B-H. Table S5). The unevenness of the DAP distribution and the excitatory-neuron-oriented regulatory pattern is consistent with the pattern we observed in the snRNA-seq dataset, providing another layer of evidence that the regulatory pattern of A;E mutant is likely to have a bigger impact on excitatory neurons in hippocampus area.

Moreover, the vast majority of DAPs show increased accessibility in A;E compared to WT (Fig. 5E), suggesting more open chromatin areas and potentially more accessible binding sites for chromatin regulatory elements in the A:E mutant. Peak annotation results show that most of the identified DAPs in IT-ET Glut and DG-IMN Glut are located very close to the transcription start site (≤1 kb) (Fig. S4I-J), suggesting a high probability in the transcription regulatory function.

To better understand the relationship between regulatory elements and their target genes, we predicted peak-to-gene links based on co- activity between gene expressions and nearby accessible chromatin regions (Table S6). In particular, we focused on IT-ET Glut population, as it represents the most affected cell type in both snRNA-seq and snATAC-seq profiles.

Among all identified peak-gene associations, two genes, *Fos* and *Cck*, drew our attention. Both genes were identified as transcriptional targets under differentially enriched regulons (*Egr1* and *Zbtb18,* respectively) in the previous GRN analysis. Gene-link-to peak analysis suggested differentially accessible peak regions were identified associated with RNA expression levels of these two genes. Furthermore, both genes are known to play roles in memory formation. Previous studies have shown that post-stimulus activation of these genes can enhance fear memory performance (Feng et al., 2021; Strekalova et al., 2003). Gene expression feature plots confirmed their co-expression patterns in IT-ET Glut cells (Fig. 5B), while snATAC-seq revealed increased chromatin accessibility near their gene-associated peak regions, with motif scanning further validating the presence of corresponding transcription factor binding sequences (Fig. 5C, D). Although both genes showed lower basal expression levels in A;E compared to WT, the increased chromatin accessibility suggest enhanced feasibility to their respective transcriptional regulators, potentially enabling a more sensitive response to environmental stimuli.

### 3.5. MERFISH verifies the transcriptional changes and enhances cell subtyping with spatial organization information

To verify the DEGs called from the 10× snMultiome assay and to explore the spatial organization of the hippocampal cells in both genotypes, we selected a panel of 300 genes for MERFISH imaging (Moffitt and Zhuang, 2016). The MERFISH gene panel comprises: (1) cell-type markers (*n* = 46) (Franjic et al., 2022; Yao et al., 2021), (2) high fold-change DEGs identified from prior 10× experiment which are biased towards neurons (*n* = 85), and (3) genes previously reported to be dysregulated in *Mecp2* mutant mice (*n* = 203) (Boxer et al., 2020; Gulmez Karaca et al., 2018). There is an overlap among the genes from the above three categories, totaling 300 genes.

3 male A;E mice and 3 male WT littermates at 9 weeks old of age were collected for MERFISH imaging (Fig. 6A). Coronal brain sections were obtained at Bregma coordinates of −2.3 ± 0.3 mm. Low-quality cells (a cell that has a transcript count of less than 50 or greater than 6000, and cell volume less than 200 or greater than 3 times of the medium) were removed from the downstream analysis (Fig. S4A).

50,409 cells were obtained for further analysis (WT/y: 26,345, A;E/y: 24,064). Batch effect was corrected with Harmony and the reproducibility between individual runs was carefully examined (Fig. S4B). Cells were clustered and major cell types were called using the same set of identifiers as for the 10× dataset (Fig. S4C). The number of unique genes per cell and the number of transcripts per cell were compared among the cell type to ensure the results are reasonable and comparable (Fig. S4D). Furthermore, a manual cell authenticity check was performed to examine the accuracy of cell segmentation. The authenticity rates in all major cell types were above 98%, suggesting a robust cell segmentation quality (Fig. S4E).

The identified and annotated cells were visualized on both UMAP (Fig. 6B) and spatial map (Fig. 6C) and colored by cell identities. All major cell types were found in both A;E and wildtype. No obvious difference in the cell type composition or cluster shifting was observed in either UMAP or the spatial map, which is similar to the observation in the snRNA-seq results (Fig. 6D). The cell pattern on the spatial map matches with the prior knowledge in anatomy.

DEGs between the two genotypes were called from each individual cell type and from the total population using 2-tailed unequal variance student *t*-test. Low expressors were removed to exclude noise (Table S7). The resulting DEGs from MERFISH platform were compared with the list from the RNA-seq. More than half of the 10×-derived DEG candidates were confirmed to be differentially expressed in the MERFISH data. Among the excitatory neuronal cell types of focus, cell type-specific predicted targets were also successfully verified: 43 out of 95 targets were validated in IT-ET Glut, and 106 out of 124 DG-IMN Glut cell candidates were confirmed (Fig. 7A-B). In particular, the transcriptional change of *Cck* was verified by MERFISH (Fig. 6G), along with several other key TGs inferred by GRN analysis, such as *Acyl2*, *Dpyd*, *Olfm3*, *Rbfox1*, *Slc25a3*, *Trpm3*, etc. Comparison between MERFISH and 10× results were made to reveal the common DEGs detected by the two independent assays (Table S8, Fig. 7D-F).

Then, we further classified the IT-ET Glut into 4 subclusters: CA1 (WT: 4238, A;E: 3713), CA2 (WT: 405, A;E: 521), CA3 (WT: 1759, A;E: 1955), and Mossy (WT: 89, A;E: 74) (Fig. 5E-F). Since these clusters are spatially well-defined in the hippocampal area, we are able validate cell clustering and annotation accuracy using spatial information. DEGs were called for each subcluster using the same method as for the major cell types. 132 DEGs were identified in the CA1 population, whereas only 3 and 18 DEGs were found in the CA2 and CA3, separately (Table S7).

To further utilize spatial information, we applied Tangram, a computational framework that can align snRNA-seq data onto spatially resolved transcriptomic data (in this case, MERFISH), and thus “project” the spatial information to the original snRNA-seq dataset. With this algorithm, we were able to add inferred spatial information to the 10× data and sub-classified the 10× IT-ET Glut cell population into 4 subtypes: CA1 (WT: 10,213 nuclei, A;E: 6210 nuclei), CA2 (WT: 1251 nuclei, A;E: 783 nuclei), CA3 (WT: 4603 nuclei, A;E: 2812 nuclei), and Mossy (WT: 312 nuclei, A;E: 183 nuclei). DEGs and DAPs were called from the subtypes. Similar to MERFISH results, more DEGs and DAPs were found in the CA1 area (DEG: 2800, DAP: 704) compared to CA2 (DEGs: 73, DAP: 25), CA3 (DEG: 612, DAP: 185), and Mossy (DEG: 3, DAP: 6) (Table S2, S5). Similarly, common DEGs between the two assays were identified. 66 out of 132 DEGs were verified in CA1 subregion (Fig. 7C), whereas 3 common DEGs were found in CA3, none were identified in CA2 or Mossy. These results suggest that the predicted IT-ET Glut targets from the prior snRNA-seq experiment may be more representative of CA1 neurons.

## 4. Discussion

Our work investigated the functional consequences of *Mecp2 S80A; S421E* double phosphor mutations through comprehensive behavioral phenotyping and single nuclei multi-omics profiling. Our findings uncover complex phenotypic patterns that cannot be simply predicted from single mutation studies and reveal a neuronal bias of modulatory role of MeCP2 phosphorylation.

Several considerations guided our experimental approach. The choice of single-nucleus sequencing was informed by prior observations. Initial whole-brain bulk RNA-seq identified few DEGs in *Mecp2*-null model (Nuber et al., 2005; Tudor et al., 2002), while region- or cell type-specific studies revealed hundreds to thousands (Boxer et al., 2020; Gulmez Karaca et al., 2018). This suggested that whole-brain approaches mask opposing or cell type-specific changes upon Mecp2 dysfunction. Our snRNA-seq strategy enabled cell type-resolved profiling, identifying substantially more DEGs than previous tissue-specific studies, further demonstrating the highly cell type-specific nature of MeCP2 regulation. While single-cell technologies are inherently noisier than bulk sequencing, we employed bioinformatic strategies, including pseudo-bulk aggregation and gene regulatory network analysis, to enhance signal robustness. Independent MERFISH validation further substantiates our key findings.

Moreover, naïve state animals were used for omics studies, despite that both pS80 and pS421 occur in an activity-dependent manner (Li and Chang, 2014). The primary reason is that both pS80 and pS421 are naturally present at detectable levels in the brains of naïve mice (Tao et al., 2009), indicating that these modifications play a role not only when undergoing behaviors, but also at the naïve state. Thus, comparing wild type versus knock-in mice with fixed phosphorylation status in the naïve condition allows us to assess the consequences of constitutive dephosphorylation/phosphorylation at S80/S421, independent of activity-induced fluctuations. Furthermore, establishing the baseline molecular phenotype in naïve animals provides a necessary foundation for future investigations. Without this baseline, it will be difficult to distinguish between intrinsic effect of fixed A;E phosphor state and those induced by behavioral induction. Moreover, performing omics analysis under naïve conditions helps minimize potential confounding factors such as stress, handling, and individual differences in behavior responses. This is especially important for *Mecp2*-related omics studies, as all transcription profiling studies on *Mecp2* mutant suggest a modest fold change level. Uncontrollable environmental variables may obscure subtle but biologically important effects. In summary, the goal of this study is to examine the functional effect of a fixed modification state that mimics constitutive dephosphorylation/phosphorylation at these two sites, as observed in active neurons. The naïve state represents a well-controlled condition for defining the core molecular signatures associated with this modification.

Through comprehensive behavioral phenotyping of the S80A;S421E double mutant, we observed the functional consequences of simultaneously disrupting two phosphorylation sites including decreased locomotion, increased central exploration in the open field, and enhanced contextual fear memory. By comparing with the previously reported phenotypes in the S80A or S421E single phosphor mutant models (Table 1), we found the combinatorial code of these two sites are not simply additive. Instead, they manifest in three distinct patterns, each suggesting different underlying mechanisms.

In assays such as running wheel, *Mecp2*^*S80A*^, *Mecp2*^*S421E*^, and *Mecp2*^*S80A;S421E*^ mice all exhibited decreased mobility. The combination of the S80A and S421E modifications appears to be consistent with the single phosphor modification outcomes. Disruption of either site alone is sufficient to impair function, and combining the mutations does not produce an additional deficit. This pattern suggests that for this particular function, S80 and S421 phosphorylation may work synergistically. However, whether the combinatorial effect is additive remains unknown. Further investigation with quantitative comparison between single and double-phosphor mutant lines is needed.

In the open field assay, we observed new phenotypes that manifest differently from either the S80A or the S421E mutant. Neither *Mecp2*^*S80A*^ nor *Mecp2*^*S421E*^ mice showed changes in the open field performance, however, *Mecp2*^*S80A;S421E*^ mice exhibit an increased central area exploration compared to wild-type littermate controls. Following elevated plus maze test and the light-dark box test denied anxiety-like phenotype, suggesting that the increased central area exploration in the open field assay may be due to other neurological features, such as spatial recognition. Still, this finding implies that the combined phosphorylation status of S80 and S421 may exert a broader regulatory impact than the simple additive effects of the individual phosphorylation sites, suggesting the existence of previously undefined crosstalk mechanisms among multiple phosphorylation sites. Another potential explanation could be molecular buffering capacity. MeCP2 operates within a complex regulatory network with inherent robustness. Individual site perturbations may be accommodated by this network through compensatory mechanisms, redundant interactions, or homeostatic adjustments. Phenotypic consequences thus remain below detection thresholds in the single phosphor mutation. However, simultaneous disruption of two sites may exceed the system's buffering capacity, unmasking latent vulnerabilities and revealing phenotypes that only emerge when multiple regulatory nodes are compromised.

Finally, in the fear conditioning assay, *Mecp2*^*S80A;S421E*^ mice exhibited enhanced contextual memory, but both *Mecp2*^*S80A*^ and *Mecp2*^*S421E*^ mice displayed decreased contextual memory. Therefore, in this assay, the direction of phenotypic modulation in the double phosphor-mutant did not align with the regulatory direction of the respective single phosphor-mutant. These findings suggest that the combinatorial code of S80 and S421 is not merely synergistic. Distinct compensatory or reorganized regulatory circuits may be engaged, therefore redirecting MeCP2's functional outputs in ways that diverge from either single phosphor modification. Although phenotypic performances of single phosphor-mutant animals cited from different studies may be confounded by different experimental designs and experimenters, this comparison provides initial context regarding potential combinatorial effect of the phosphorylation statuses at MeCP2 S80 and S421.

Utilizing the single-cell resolution multiome techniques, we observed a cell type-specific regulatory pattern biased towards neuronal populations, especially the glutamatergic excitatory neurons, suggesting that phosphorylation status at S80 and S421 sites predominantly regulate excitatory neuronal functions. The observation is also consistent with established prior knowledge indicating that the phosphorylation events at S80 and S421 are dynamically modulated by neuronal activity (Damen and Heumann, 2013; Tao et al., 2009).

In addition, single-cell resolution profile allows us to conduct secondary analysis and predict alterations on gene regulatory network (GRN), which is critical for our understanding of MeCP2 function. The GRN analysis revealed cell-type specific differentially enriched regulatory subnetworks between A;E and WT conditions, highlighting transcription factors and associated target genes that may play a role in the altered hippocampal functions. snATAC-seq data confirmed the altered chromatin accessibility at the TG-expression-associated region with the presence of TF binding motif. Collectively, these results point to potential molecular mechanisms underlying altered hippocampal functions in the A;E mutant. However, the fold changes of these target genes are modest, consistent with previously published *Mecp2* sequencing studies reporting broad yet mild transcriptional changes (Boxer et al., 2020; Gulmez Karaca et al., 2018). Such subtle and highly specific changes pose challenges for experimental validations and complicate the determination of whether specific pathway changes represent the primary drivers of functional alterations, or whether all affected genes contributed partially. Still, the resulting list of candidate regulatory subnetworks and interaction pairs inferred by CCI provides a valuable resource and direction for subsequent research. This finding underscores the significant role of MeCP2 in the global transcription regulation and highlights the importance of investigating cellular and intercellular communication in future studies. Together, our single cell resolution data suggested a cell-type specific regulatory role of *Mecp*^*2S80A;S421E*^, which may be overlooked by tissue-level examinations.

Another intriguing observation from the snMultiome result is the seemingly dissociation between chromatin accessibility changes and transcriptional outcomes. While we observed widespread increases in chromatin accessibility, which suggested more open binding sites for chromatin regulatory elements, the transcriptional changes didnť exhibit any dominant trend, and the gene expression fold change remains modest. Several biologically rational mechanisms may explain this decoupling.

Firstly, the relationship between chromatin accessibility and gene expression is not a simple positive correlation. Transcriptional repressor motifs and repressor binding sites can act in concert with activator domains to form complex regulatory networks. In addition, post-transcriptional regulatory mechanisms may compensate for altered chromatin states. Therefore, increased chromatin accessibility does not necessarily translate directly into elevated transcription, particularly in the absence of appropriate cellular signals or transcription factor activation.

Secondly, increased chromatin accessibility does not equate to increased binding of regulatory elements. As shown by our findings on *Fos* and *Cck*, the two genes exhibited enhanced accessibility near their gene-associated peak regions yet showed lower basal expression levels in the A;E mutant compared to WT controls. This apparent dissociation may reflect a poised chromatin state that facilitates rapid responsiveness rather than sustained upregulation, enabling a more sensitive response to environmental stimuli. Furthermore, the relatively low basal expression levels may serve to maintain baseline function while allowing a wider dynamic range between the basal and activated states. Such a configuration could be functionally advantageous, potentially enhancing the magnitude of transcriptional induction upon stimulation and thereby contributing to improved memory performance.

Collectively, these observations suggest that in the A;E mutant, widespread chromatin opening may reflect a primed epigenetic landscape that preferentially prepares specific genes for inducible expression, rather than driving constitutive transcriptional changes. Such dissociation between accessibility and expression underscores the value of integrating both epigenetics and transcriptional dimensions when interpreting regulatory dynamics in complex biological systems. More broadly, these considerations highlight the importance of integrating multi-omic approaches to fully capture the regulatory consequences of MeCP2 modifications.

To independently validate our transcriptional findings, we employed MERFISH as an independent imaging-based platform. 43 out of 95 (45%) DEGs were validated in the IT-ET Glut neuronal population, and 106 out of 124 (85%) were validated in the DG-IMN Glut population. Key transcriptional targets predicted by GRN analysis, such as Cck, were validated as well. These concordance rates, particularly the high validation in DG neurons, strengthen confidence in our core findings.

Nonetheless, the concordance rates between snRNA-seq and MERFISH warrant further discussion. Discrepancies between platforms are not uncommon and can arise from multiple factors. First, the two technologies are fundamentally different: snRNA-seq is sequencing-based, while MERFISH is imaging-based, leading to inherent differences in detection sensitivity and dynamic range. Second, the spatial sampling strategies differ. snRNA-seq libraries were generated from whole hippocampal lysates, pooling nuclei across the entire rostral-caudal axis. In contrast, MERFISH was performed on coronal brain sections restricted to specific rostral-caudal locations, which may not capture the full spatial heterogeneity of gene expression in the entire hippocampus. These technical and sampling differences are likely to contribute to the observed variability. Despite these challenges, our study provides a direct comparison between two emerging omics technologies. This side-by-side evaluation offers valuable insights into cross-platform data interpretation and may inform future experimental design in the field.

Our findings contribute to a growing body of literature characterizing the functional roles of specific MeCP2 phosphorylation sites. In addition to the S80A, S421A;S424A, and S421E mutations already cited in the manuscript (Hongda Li, 2013; Li et al., 2011, 2014; Tao et al., 2009; Zhong et al., 2019), other phosphorylation sites have been functionally characterized. For instance, Ebert et al. reported that phosphorylation at T308 disrupts MeCP2's interaction with the nuclear receptor co-repressor (NCoR) complex (Ebert et al., 2013). Similarly, Gonzales et al. (2012) demonstrated that pS229 is involved in modulating MeCP2's interaction with heterochromatin (Gonzales et al., 2012). These findings, together with our own, underscore the complexity of MeCP2 regulation through multiple phosphorylation events.

Our demonstration that dual phospho-site mutations produce synergistic, opposing, and emergent phenotypes adds an important layer to current understanding. It suggests that the regulatory functions encoded by MeCP2 phosphorylation are not merely additive but combinatorial, with site combinations generating unique functional states. This combinatorial complexity may be particularly relevant in neurons, where dynamic, activity-dependent phosphorylation at multiple sites could enable graded and context-specific regulation of MeCP2 function. The complexity of MeCP2 regulation through combinatorial post-translational modifications may help explain the pleiotropic effects of MECP2 mutations in Rett syndrome and refresh the view of simple genotype-phenotype correlations.

One limitation of our study is that the data are mostly correlative. Future work should focus on establishing the causal relationship between the identified pathways and the observed behavior and electrophysiological alterations in the A;E mice, which would substantially enhance our knowledge in the regulatory functions of Mecp2 phosphorylation. Nonetheless, our finding underscores the relevance of the pathway findings as a foundation for future mechanistic studies.

In summary, our study demonstrates that combined *Mecp2* S80A; S421E phosphorylation mutations produce complex phenotypic outcomes. At the molecular level, we observe glutamatergic-neuronal-oriented transcriptional and chromosomal changes in *Mecp2* S80A; S421E hippocampus. Our use of single-nucleus multi-omics and independent MERFISH validation provides cell type-resolved insights into MeCP2 function and provide high-quality data and valuable cross-platform comparison. These findings advance our understanding of how combinatorial post-translational modifications tune MeCP2 function and highlight the importance of studying multi-site regulation in neurodevelopmental disorders.

## Supplementary Material

MMC10

mmC11

MMC12

MMC13

MMC14

MMC15

MMC16

MMC7

MMC8

MMC9

MMC1

MMC3

MMC5

MMC4

MMC2

MMC6

Supplementary data to this article can be found online at https://doi.org/10.1016/j.nbd.2026.107521.

## Figures and Tables

**Fig. 1.**
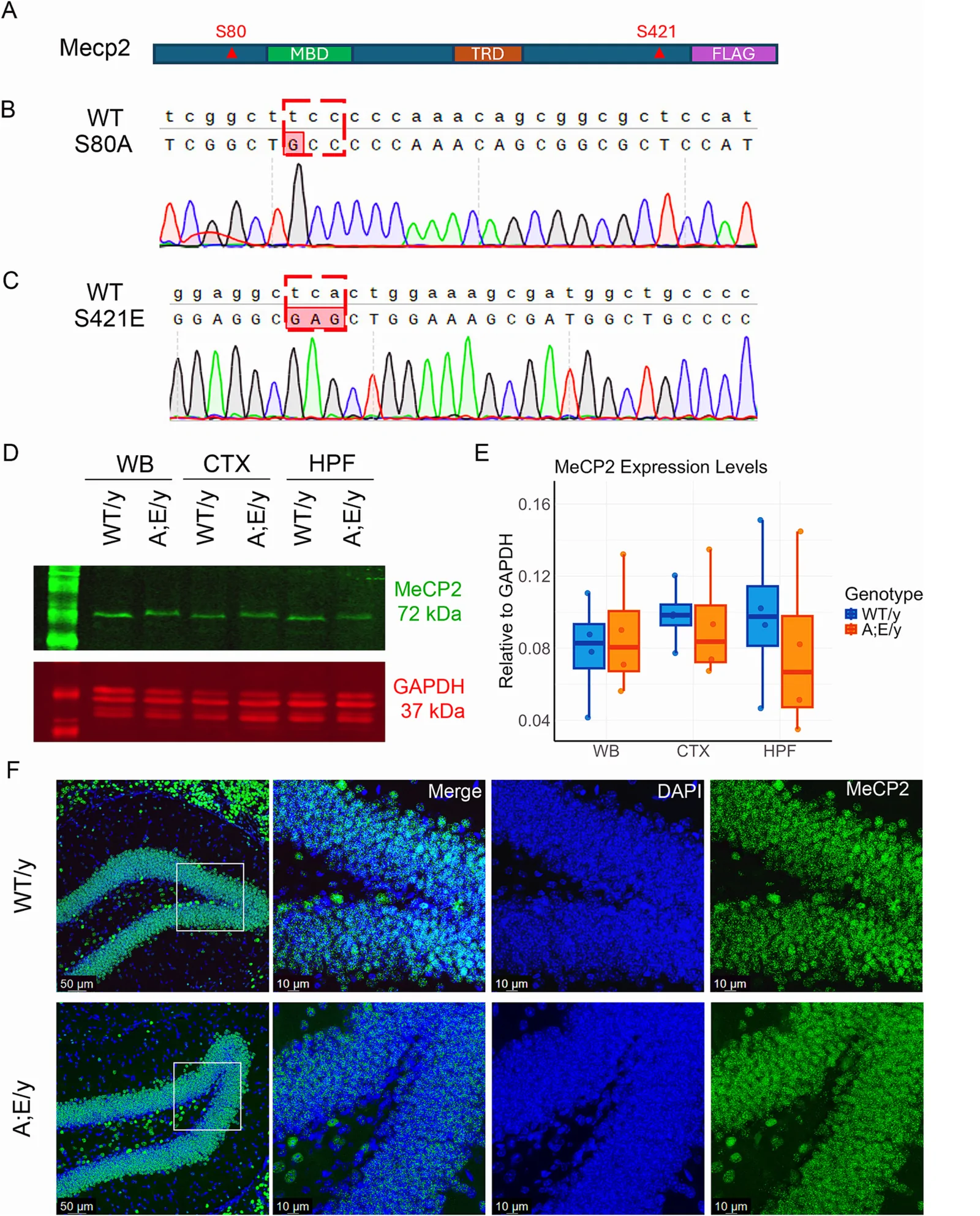
Mecp2 S80A;S421E mutant mouse line generation. A) Sketch of Mecp2 gene and mutation sites. Point mutation validated by sequencing at S80 (B) and S421 (C). D) Representative Western Blot image showing there's no change in the overall MeCP2 protein expression level in the brain. E) Box plot showing the quantification results of the total MeCP2 expression levels relative to GAPDH in different brain regions in WT/y and A;E/y animal. The box represents the data between 25% and 75% percentile. The midline inside the box represents the median. No significant effect was detected in genotype (F(1,18) = 0.18, *p* = 0.676), region (F(2,18) = 0.24, *p* = 0.787), or genotype × region interaction (F(2,18) = 0.32, *p* = 0.733). The whiskers represent 1.5 times of the interquartile range extended from the top and the bottom of the box. Super-imposed scatters represent independent biological repeats. WB: whole brain; CTX: cortex; HPF: hippocampus. F) Representative immunofluorescent staining of MeCP2 and DAPI at the dentate gyrus area of WT/y and A;E/y.

**Fig. 2.**
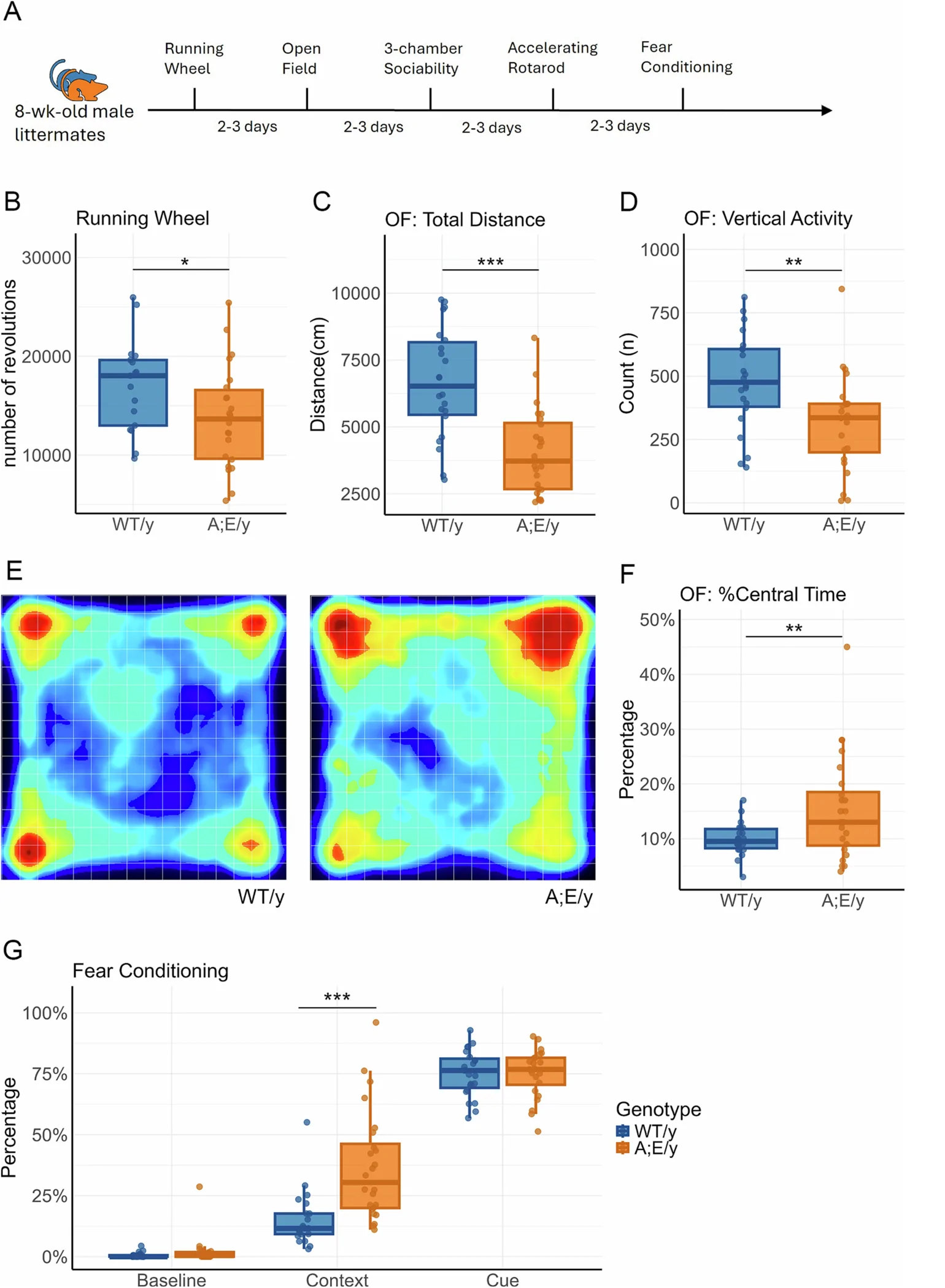
Behavioral phenotyping of Mecp2 A;E/y mice. A) Timeline of behavioral assays, ordered by stress from low to high. RW: running wheel; OF: open field; SOC: 3-chamber sociability test; AR: accelerating rotarod; FC: fear conditioning. B) Box plot showing the total running distance during the dark cycle running wheel test. N(WT) = 17, N(AE) = 22; *p* = 0.050 (student *t*-test). C) Box plot showing the total distance traveled during the Open Field assay. N(WT) = 22, N(AE) = 23; *p* = 3.177 × 10^−5^ (student t-test). D) Box plot showing the vertical activity counts during the Open Field assay. *p* = 2.269 × 10^−5^ (student t-test). E) Representative heat maps of individual WT/y (left) and A;E/y (right). F) Box plot showing the percentage of time spent exploring the central area in the open field assay. N(WT) = 22, N(AE) = 23; *p* = 0.032 (student t-test). G) Box plot showing the results of the fear conditioning assay. N(WT) = 24, N(AE) = 25; p(baseline) = 0.211, p(context) = 9.61 × 10^−5^, p(cued) = 0.979 (repeated measures ANOVA). All statistical details can be found in Table S1. In all the box plots, the box represents the data between 25% and 75% percentile. The midline inside the box represents the median. The whiskers represent 1.5 times of the interquartile range extended from the top and the bottom of the box. Super-imposed scatters represent independent biological repeats.

**Fig. 3.**
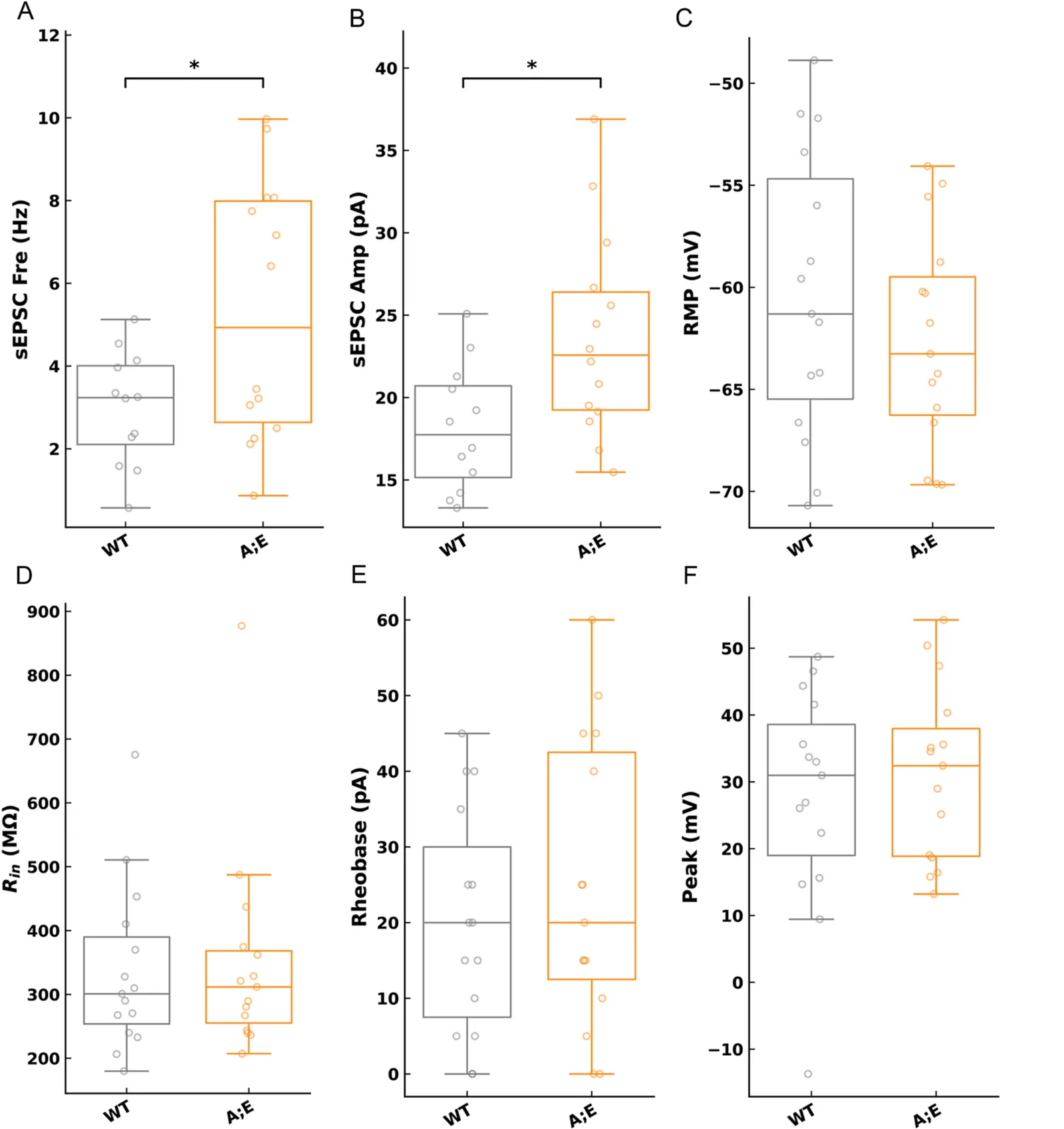
Summary of results from whole-cell patch-clamp recording experiments. Whole-cell patch-clamp recording of CA1 pyramidal neurons were collected from acute hippocampal slices from four pairs of adult wild type and AE mice. Box plots showing A) sEPSC frequency. N(WT) = 12, N(AE) = 14; *p* = 0.021 (Welch's t-test); B) sEPSC amplitude. N(WT) = 12, N(AE) = 14; *p* = 0.011 (Welch's t-test); C) Resting Membrane Potential (RMP). N(WT) = 15, N(AE) = 15; *p* = 0.342 (unpaired two-tailed Studenťs t-test); D) Input Resistance (R_in_). N(WT) = 15, N(AE) = 15; *p* = 0.868 (unpaired two-tailed Studenťs t-test); E) Rheobase. N(WT) = 15, N(AE) = 15; *p* = 0.461 (unpaired two-tailed Studenťs t-test); and F) Spike peak. N(WT) = 15, N(AE) = 15; *p* = 0.534 (unpaired two-tailed Studenťs t-test).

**Fig. 4.**
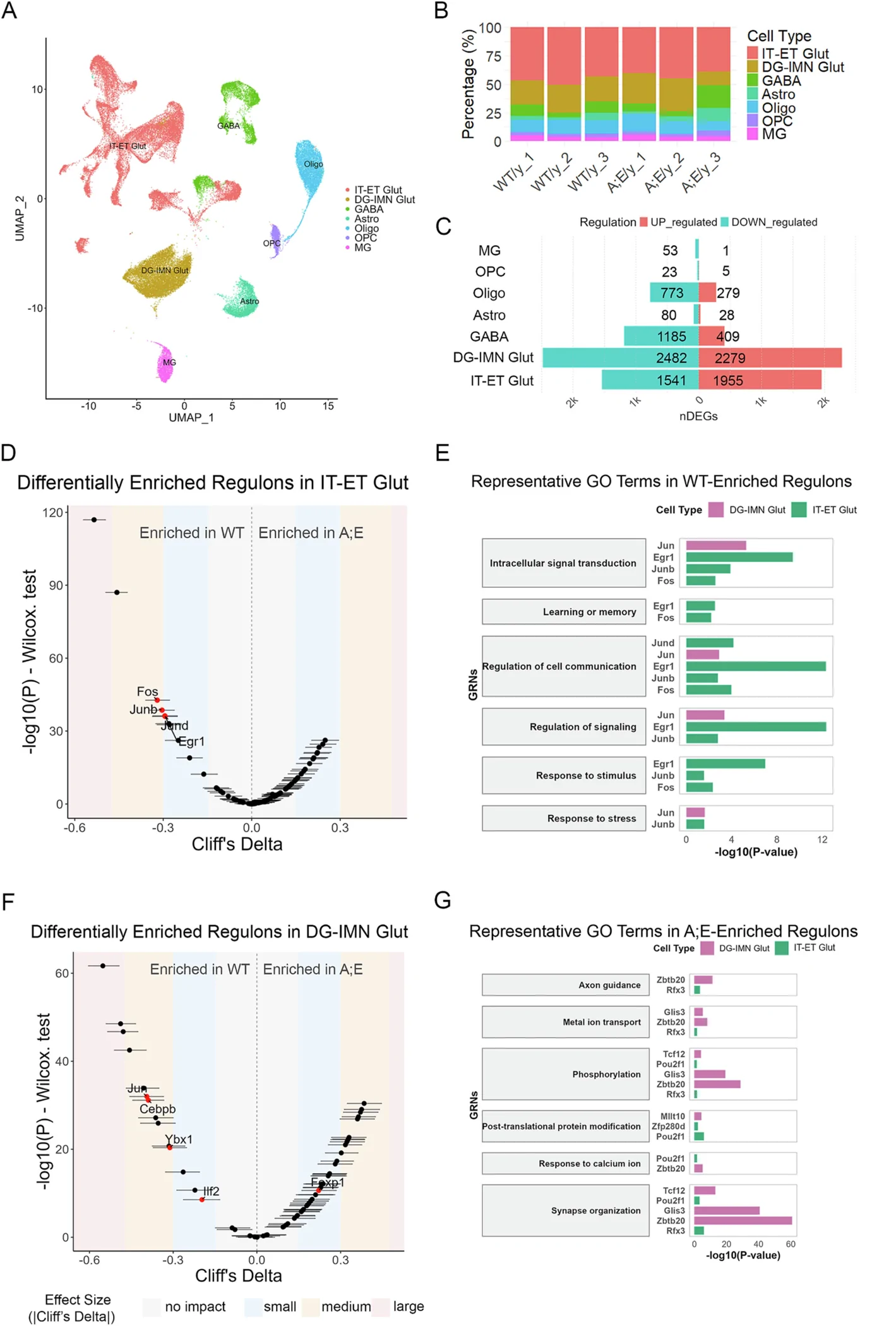
snRNA-seq data of WT/y and A;E/y mouse hippocampi. A) snRNA-seq based UMAP visualization of major cell classes. B) Accumulated bar graph showing the cell type composition in each sample. C) Diverging bar graph showing the numbers of DEGs in each cell type. D, F)Volcano plot showing the differentially enriched GRNs in the (D) IT-ET Glut and (F) DG-IMN Glut populations. Y-axis represents the -log10(p) from the Wilcoxon rank sum test comparing A;E and WT, and x-axis represents the Cliff's delta effect sizes. E,G)Horizontal bar graphs showing the enriched GO terms favoring (E) WT and (G) A;E populations.

**Fig. 5.**
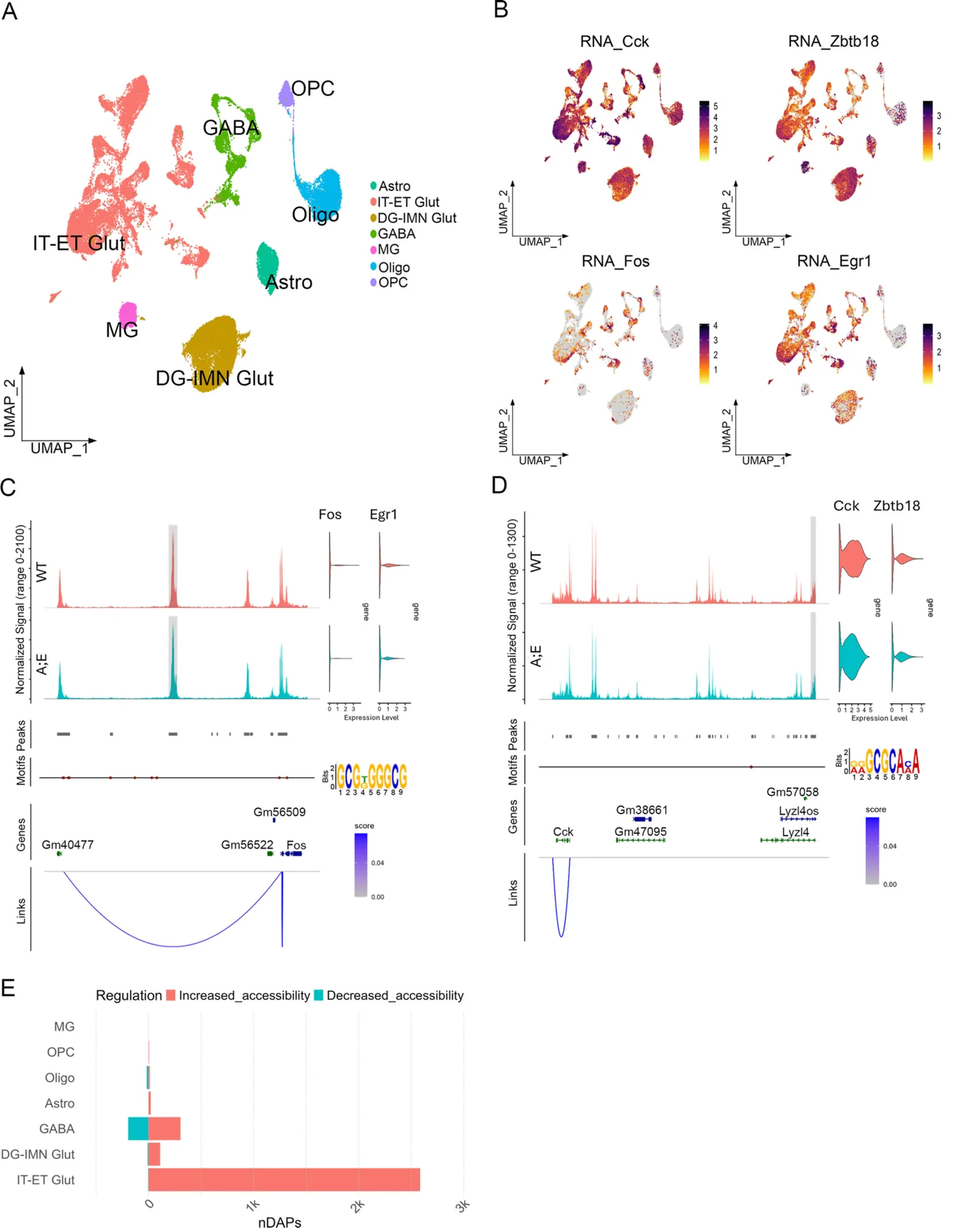
snATAC-seq data of WT/y and A;E/y mouse hippocampi. A) Integrated UMAP visualization of major cell classes. B) Feature plots showing the gene expression patterns of *Cck, Zbtb18, Fos*, and *Egr1* in all cell types. C) Coverage plot showing *Fos* has differential gene expressions, differentially accessible peaks, correlated gene expression levels, and *Egr1* motif binding sites in the IT-ET Glut. D) Coverage plot showing *Cck* has differential gene expressions, differentially accessible peaks, correlated gene expression levels, and *Zbtb18* motif binding sites in the IT-ET Glut. E) Diverging bar graph showing the numbers of DAPs in each cell type.

**Fig. 6.**
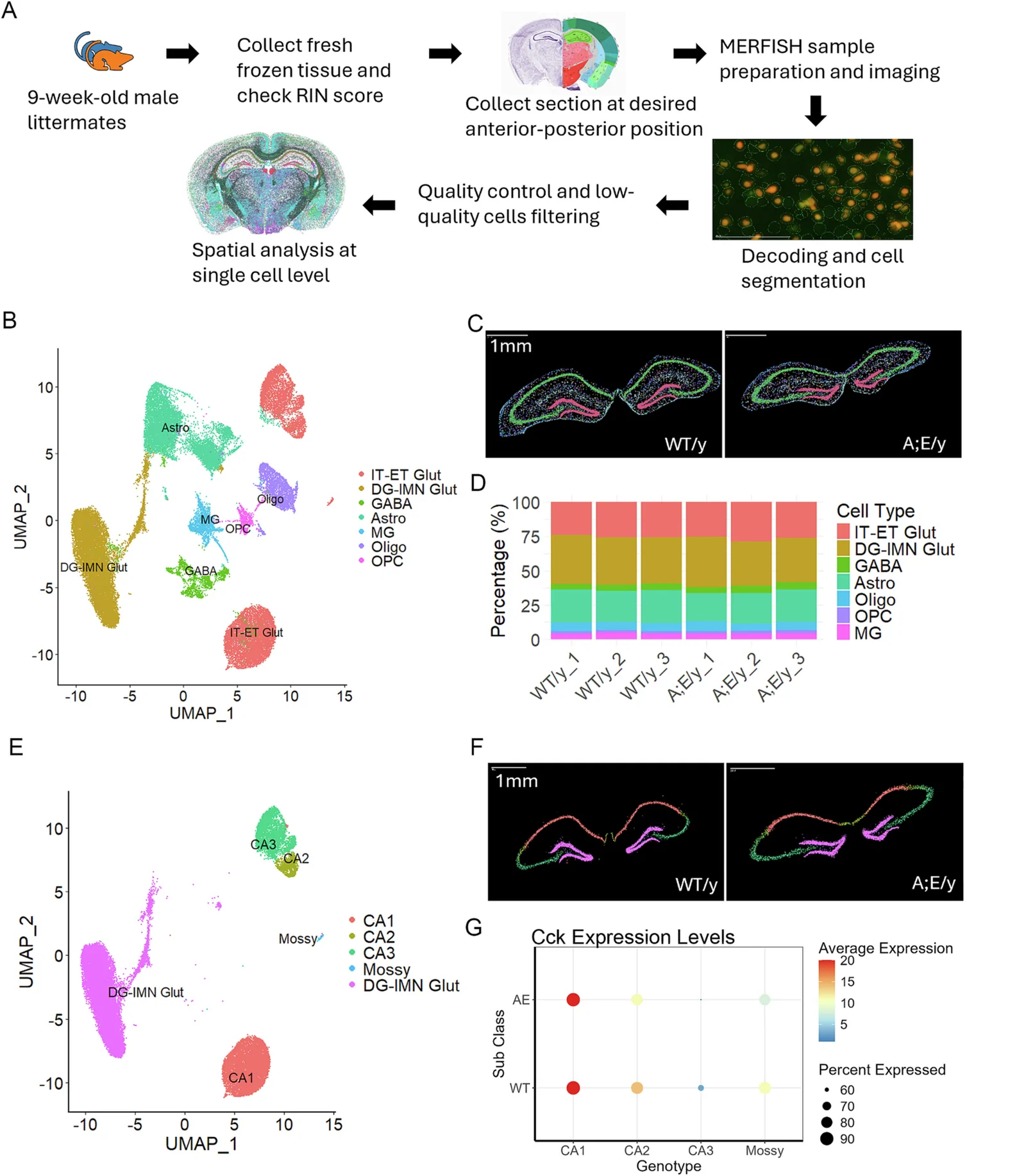
MERFISH data of WT/y and A;E/y mouse hippocampi. A) Workflow of MERFISH profiling on mouse hippocampus, including sample collection, sample preparation, MERFISH imaging, data processing, and data analysis. B, C)Visualization of all major cell clusters obtained through MERFISH data in (B) UMAP and (C) spatial map. D) Accumulated bar graph showing the composition of the major cell types in count/area size density. E, F) Visualization of sub-clustering of glutamatergic cell populations in (D) UMAP and (F) spatial map. G) Bubble plot showing the *Cck* gene expression levels measured by MERFISH in IT-ET Glut sub regions.

**Fig. 7.**
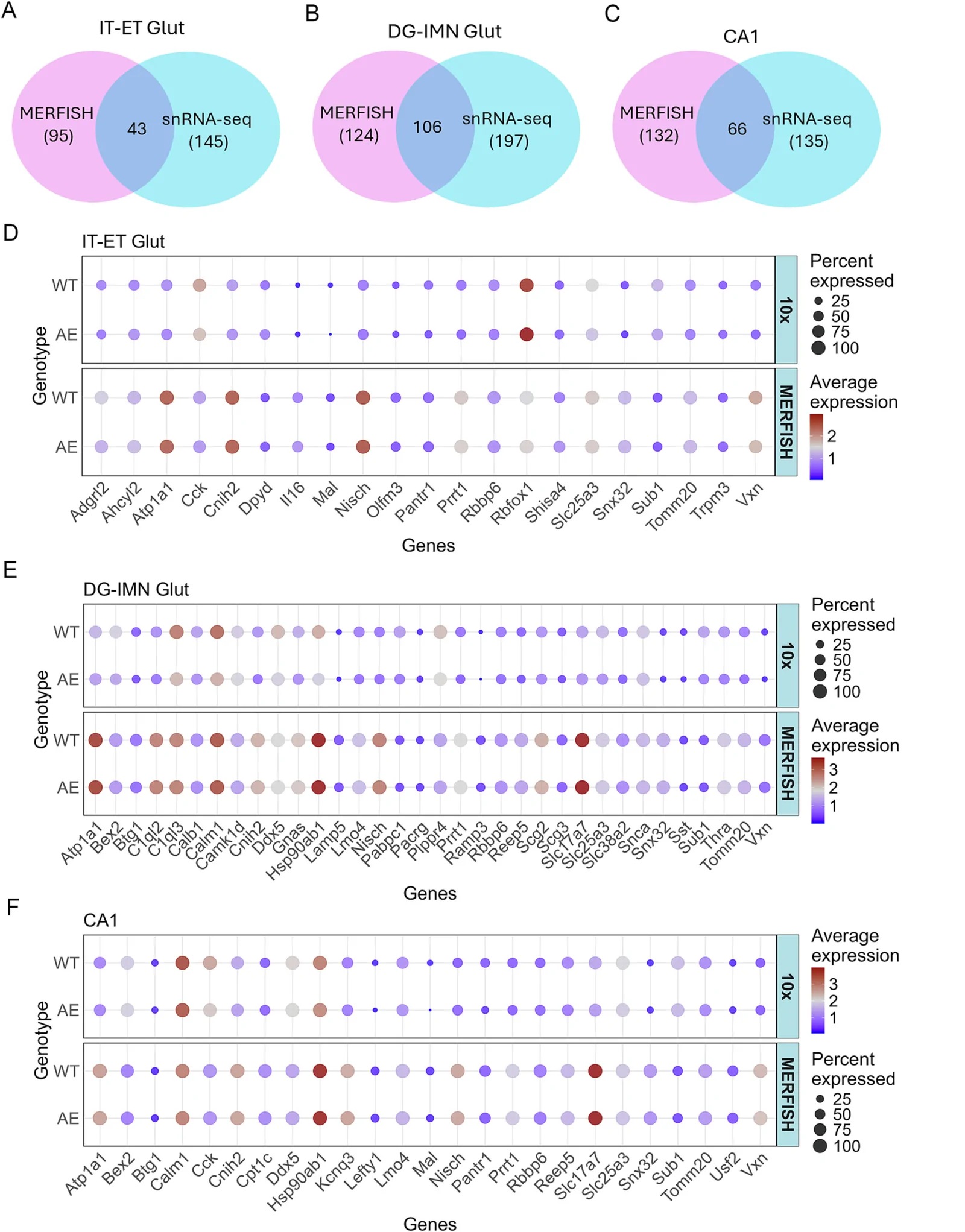
Validated DEGs between 10× and MERFISH platforms. A-C)Venn graphs showing the overlapping status between MERFISH and 10× identified DEGs in IT-ET Glut (A), DG-IMN Glut (B), and CA1 (C) cell populations. Dot plots showing the expression levels of common DEGs detected by 10× and MERFISH platforms in D) IT-ET Glut, E) DG-IMN Glut, and F) CA1 cell populations.

**Table 1:** Phenotypic comparisons between *Mecp2 S80A, S421E*, and *S80A;S421E* mutations.

Assay	*Mecp2^S80A^*	*Mecp2^S421E^*	*Mecp2^S80A;S421E^* (this work)

Running Wheel	Decreased locomotion (Tao et al., 2009)	Trend of decrease locomotion (Hongda Li, 2013)	Decreased locomotion
Open Field	No change (Hongda Li, 2013)	No change (Hongda Li, 2013)	Increased central exploration
Fear Conditioning	Decreased contextual fear memory (Tao et al., 2009)	Decreased contextual fear memory (Zhong et al., 2019)	Enhanced contextual fear memory

Comparison of the common assays performed across *Mecp2 S80A, S421E*, and *S80A;S421E* mutations mouse models.

## Data Availability

scMultiome data accession number: GSE333915.
